# PPAR-γ integrates obesity and adipocyte clock through epigenetic regulation of *Bmal1*

**DOI:** 10.7150/thno.69054

**Published:** 2022-01-16

**Authors:** Shuai Wang, Yanke Lin, Lu Gao, Zemin Yang, Jingpan Lin, Shujing Ren, Feng Li, Jing Chen, Zhigang Wang, Zhiyong Dong, Pinghua Sun, Baojian Wu

**Affiliations:** 1 Institute of molecular rhythm and metabolism, Guangzhou University of Chinese Medicine, Guangzhou 510405, China.; 2 College of Pharmacy, Jinan University, Guangzhou 510632, China.; 3 The First Affiliated Hospital of Jinan University, Guangzhou 510632, China.

**Keywords:** PPAR-γ, obesity, circadian clock, BMAL1, SLC1A5

## Abstract

While growing evidence suggests that circadian clock and obesity are intertwined, the underlying mechanism is poorly understood. Here, we investigate how circadian clock is linked to obesity.

**Methods:** Metabolomics profiling of WAT (white adipose tissue) samples was performed to identify the metabolites altered in obese model. mRNA levels were analyzed by qPCR assays. Proteins were detected by immunoblotting, immunofluorescence and ELISA. ChIP and luciferase reporter assays were used to investigate epigenetic and transcriptional regulation.

**Results:** Obesity causes perturbance of circadian clock in WAT in mice and humans, particularly, BMAL1 is markedly reduced. Metabolomic analysis reveals reduced glutamine and methionine in obese WAT. Glutamine metabolism contributes to production of acetyl-CoA, whereas methionine metabolism generates S-adenosyl methionine (SAM). Acetyl-CoA and SAM are the substrates for histone acetylation and methylation, respectively. Reduced glutamine and methionine in obese WAT are associated with decreased H3K27ac and H3K4me3 at *Bmal1* promoter. Consistently, glutamine or methionine administration *in vitro* and *in vivo* increases H3K27ac or H3K4me3, promoting *Bmal1* transcription and expression. A screen of transport and metabolic genes identifies downregulation of the uptake transporter SLC1A5 as a cause of reduced glutamine or methionine in obese WAT. Moreover, we observe impaired expression of PPAR-γ in obese WAT. PPAR-γ trans-activates *Slc1a5* via direct binding to a response element in promoter.

**Conclusion:** Impaired PPAR-γ in obesity provokes downregulation of SLC1A5 and reductions in adipocyte uptake of glutamine and methionine (two epigenetic modulators), leading to disruption of *Bmal1*. Therefore, PPAR-γ integrates obesity and adipocyte clock, promoting a vicious cycle between circadian disruption and obesity development.

## Introduction

Obesity (defined as abnormal fat accumulation in the body) has become a major public health problem, affecting a growing number of countries. Currently, more than 650 million adults (about 13% of the population) in the world are obese, which has nearly tripled since 1975 1. Of note, 33% of Americans are obese, and the prevalence may hit 50% by 2030 2. Obesity is closely associated with metabolic complications (e.g., insulin resistance, glucose intolerance, hyperlipidemia, hypertension and fatty livers) and is a risk factor for several deadly diseases, including heart disease, stroke, diabetes and cancers 1,3. Contributing factors, both genetic and environmental, have been proposed for obesity development, and a combination of excess calorie intake and sedentary lifestyle is regarded as the primary cause 4. However, calorie restriction coupled with exercise has been largely ineffective in maintaining long-term weight loss, and the obesity pandemic continues unabated 5. Therefore, there is a desperate need for novel insights into the mechanisms controlling obesity and energy homeostasis.

Circadian clocks orchestrate internal daily (~24 h) rhythms of molecular and cellular processes, leading to circadian variations in physiology and behaviors (e.g., body temperature, heartbeats, feeding behavior and sleep) 6. In mammals, the clock located in the suprachiasmatic nucleus (SCN) of the brain acts as the central pacemaker coordinating the clocks present in other regions of the brain and in peripheral tissues 7. These clocks are cell-autonomous oscillators built on several interconnected feedback loops 8. In the core loop, the transcription factors brain and muscle ARNT-like protein-1 (BMAL1) and circadian locomotor output cycles kaput (CLOCK) associate to form a heterodimer that activates the transcription of clock-controlled genes (CCGs), including periods (*PERs*) and cryptochromes (*CRYs*) 9. As PER and CRY proteins accumulate, they translocate into the nucleus and inhibit the transcriptional activity of BMAL1/CLOCK complex, thereby down-regulating the expression of CCGs in a negative feedback manner 8. This feedback mechanism generates ~24 h oscillations in circadian gene expression 10. Extra feedback loops involve the key players REV-REBs, RAR-related orphan receptors (ROR)s, and D-box acting proteins [e.g., albumin D-site-binding protein (DBP) and E4 promoter-binding protein 4 (E4BP4)] that serve to maintain robustness of the clock system by regulating BMAL1 and PER2 expression 8. In addition, circadian oscillations can be modified by post-transcriptional mechanisms such as phosphorylation of clock proteins by casein kinases 11.

Disruptions of circadian rhythms, by either genetic or environmental factors, enhance the risk of obesity and metabolic syndrome 12,13-14. For instance, *Clock* and *Bmal1* mutant mice are obese prone and develop metabolic syndrome 12,15,16. Social jetlag and shift work (measures of circadian desynchrony in humans) are associated with metabolic disturbances and obesity 14,17. The mechanisms for circadian dysfunction-induced obesity are complex and multifaceted, including promotion of insulin resistance, inducement of leptin resistance, altered adipogenesis and attenuated feeding rhythm (or increased food intake in rest period) 18-21. On the other hand, people who are overweight or obese show altered expression of clock genes 22,23. Likewise, disruptions of clock genes and circadian behaviors are noted in obese mice 24-26. For instance, *Bmal1*, *Clock* and *Per2* are altered in fat and liver tissues of high-fat fed mice 27. These suggest that obesity and circadian system are tightly intertwined (a crosstalk phenomenon), which perhaps is involved in the pathogenesis of obesity and is a contributing factor to the obesity epidemic. However, the molecular mechanisms underlying the crosstalk are poorly defined. In particular, it remains to be answered how circadian clock is linked to obesity, which forms an essential aspect of the clock-obesity crosstalk.

PPARs (peroxisome proliferator-activated receptors) are a family of ligand-responsive nuclear receptors that contain three members, namely, PPAR-α, PPAR-β (also known as PPAR-δ) and PPAR-γ. PPAR family members can be activated by endogenous ligands such as fatty acids and their metabolites 28. Upon ligand binding, PPARs form heterodimers with retinoid X receptors and bind to a specific DNA response element (PPRE) to regulate gene transcription and expression 28. PPAR isoforms show tissue-specific differences in their expression and functions 28. Notably, PPAR-γ is predominantly present in adipose tissues and regarded as a master regulator of adipogenesis and lipid metabolism 29. Downregulation of PPAR-γ has been noted in obese mice 27,30. Mutations in* PPAR-γ* gene lead to partial lipodystrophy in humans 31,32. In addition, PPAR-γ has a critical role in regulating insulin sensitivity, inflammation, tumor cell growth, apoptosis and differentiation 33. These specific and crucial functions render PPAR-γ an attractive drug target for metabolic diseases and cancers 34,35. In fact, the PPAR-γ agonists rosiglitazone and pioglitazone are clinically used as insulin-sensitizing drugs for management of type 2 diabetes.

Here, we show disrupted expression of clock genes in white adipocyte tissue in obese mice and humans, and altered circadian behaviors (feeding rhythm) in obese mice. Mechanistically, PPAR-γ, downregulated in obesity, provokes changes in a cascade of events in the adipocytes, including decreased expression of the uptake transporter SLC1A5, reductions in glutamine and methionine uptake, lower production of acetyl-CoA and S-adenosylmethionine (SAM), and reduced histone acetylation and methylation, thereby repressing *Bmal1* transcription and expression. We thus propose that PPAR-γ integrates obesity and adipocyte clock via epigenetic regulation of *Bmal1*, promoting a vicious cycle between circadian disruption and obesity development.

## Results

### Disruption of adipocyte clock in obese mice and humans

**Figure 1:**
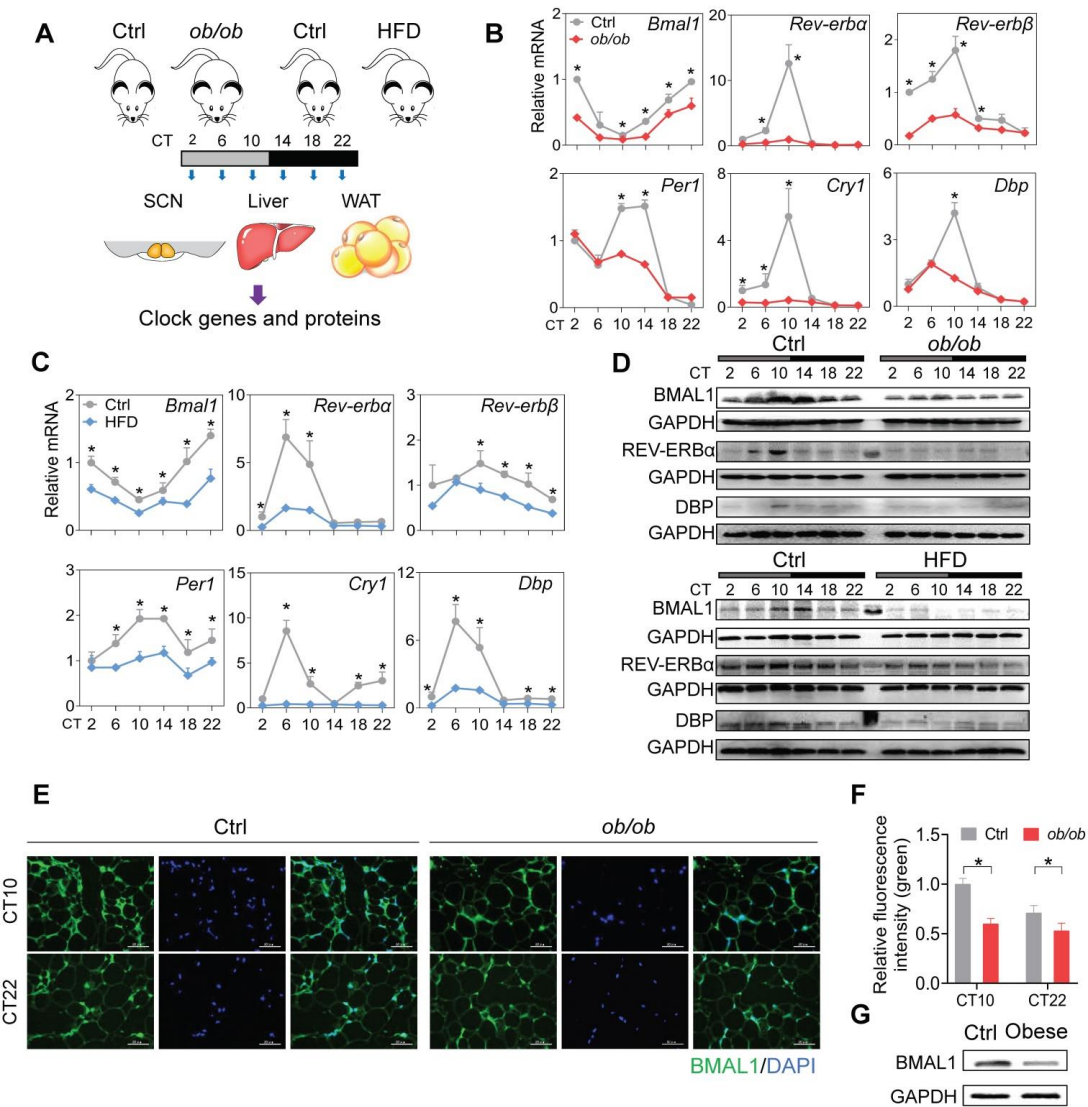
** Disruption of adipocyte clock in obese mice and humans. (A)** Schematic diagram of the experimental design for collecting SCN, liver, and WAT from *ob/ob*, high-fat diet (HFD) and control mice. **(B)** mRNA expression of clock genes (*Bmal1*, *Rev-erbα*, *Rev-erbβ*, *Per1*, *Cry1* and *Dbp*) in WAT derived from *ob/ob* and control mice at 6 circadian times (CT2, CT6, CT10, CT14, CT18 and CT22). CT, Circadian time. Data are mean ± SD (*n* = 5). *p < 0.05 at individual time points as determined by two-way ANOVA and Bonferroni post hoc test.** (C)** mRNA expression of clock genes (*Bmal1*, *Rev-erbα*, *Rev-erbβ*, *Per1*, *Cry1* and *Dbp*) in WAT derived from HFD and control mice at 6 circadian time points. Data are mean ± SD (*n* = 5). *p < 0.05 at individual time points as determined by two-way ANOVA and Bonferroni post hoc test.** (D)** Protein expression of BMAL1, REV-ERBα and DBP in WAT derived from *ob/ob*, HFD and control mice at 6 circadian times. The middle lane is the pre-stained protein ladder. **(E)** Immunostaining of BMAL1 protein (green) in WAT derived from *ob/ob* and control mice at CT10 and CT22. Scale bar = 50 μm. **(F)** Quantification of green fluorescence intensity in Fig 1E. The fluorescence intensity relative to control (CT10) is shown and expressed as mean ± SD. **(G)** Protein expression of BMAL1 in omental WAT derived from obese patients (*n* = 13) and lean individuals (*n* = 10). The samples for each group were pooled for Western blotting.

To test the effects of obesity on circadian rhythms, we used two mouse models, namely, *ob/ob* mice and mice with high-fat diet (HFD, 60% kcal from fat)-induced obesity. The two mouse models of obesity were validated by measuring adipocyte diameter and circulating lipid levels (Figure S1). We examined the expression of clock genes in SCN, WAT (white adipose tissue) and liver from the obese and control mice at six circadian times (CT2, CT6, CT10, CT14, CT18 and CT22) (Figure 1A). Obesity resulted in overall disruption of core clock genes (i.e., *Bmal1*,* Clock*,* Rev-erbα/Nr1d1*, *Rev-erbβ/Nr1d2*, *Per1*, *Per2, Cry1*, *Cry2*, *Dbp* and *Rorα*) in WAT (Figure 1B-C & Figure S2). Notably, most clock genes were markedly downregulated, including *Bmal1* and its direct targets *Rev-erbα*/*β*, *Per1*/*2, Cry1*/2, *Dbp* and *Rorα* (Figure 1B-C & Figure S2) 36,37. This suggests a crucial role of *Bmal1* in connecting obesity to adipocyte clock machinery. Consistent with mRNA changes, BMAL1, REV-ERBα and DBP proteins were downregulated in WAT of obese mice and their circadian rhythms were blunted (Figure 1D). Deregulation of circadian WAT BMAL1 was further confirmed by immunofluorescence staining (Figure 1E-F). By contrast, core clock genes such as *Bmal1*, *Clock*, *Rev-erbα* and *Dbp* were unaffected in SCN, and slightly altered in the liver in obese mice compared to control mice (Figure S3). In addition, downregulation of BMAL1 was observed in the omental WAT of obese patients as compared to lean individuals (Figure 1G). Altogether, these findings indicate that obesity is associated with clock dysfunction in WAT.

### Altered circadian behaviors in obese mice

**Figure 2:**
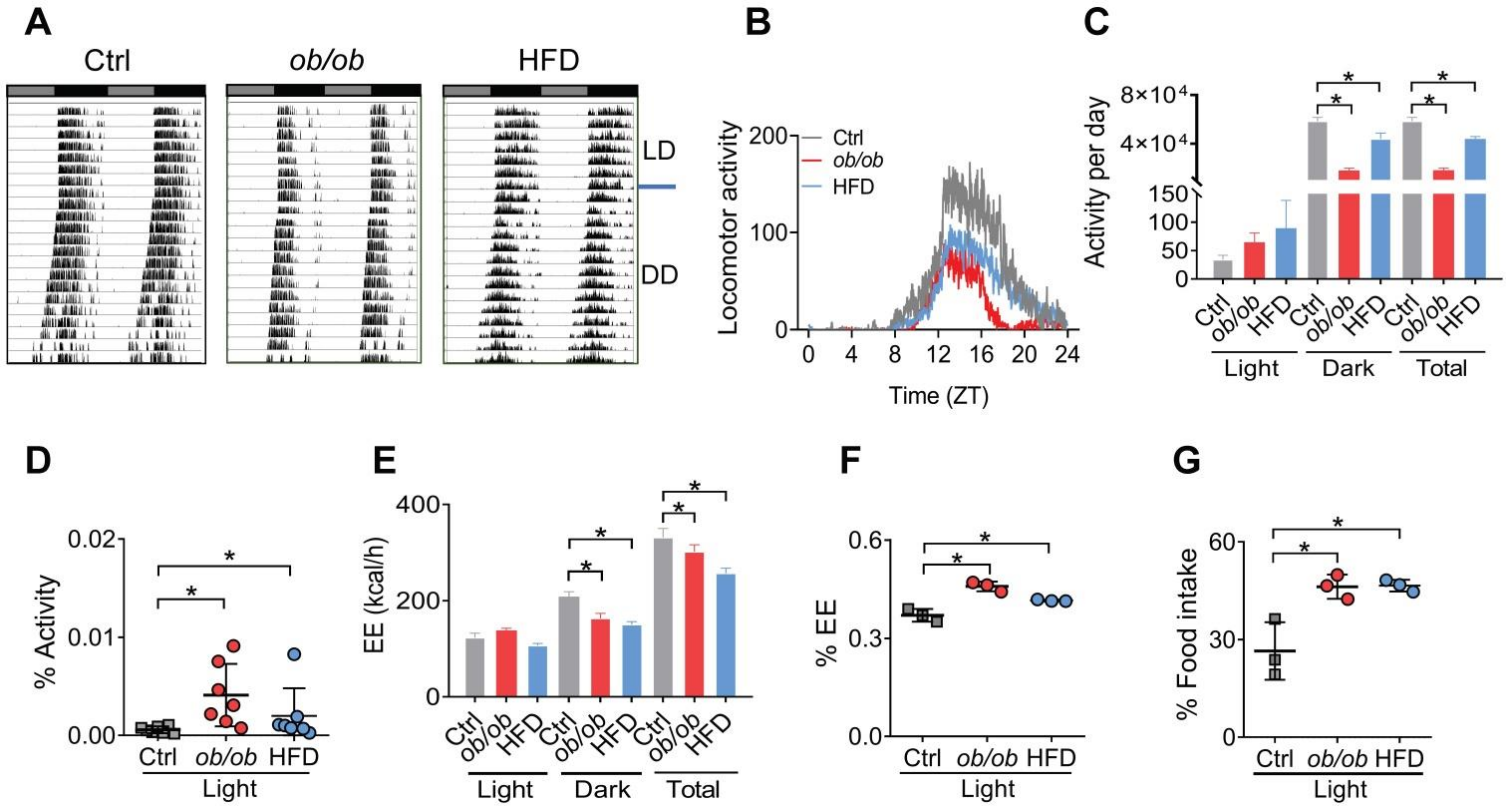
** Altered circadian behaviors in obese mice*.* (A)** Representative double-plotted actograms for daily locomotor activities of control (left panel), *ob/ob* (middle panel) and HFD (right panel) mice. Horizontal black and white bars at the top of each actogram represent (subjective) light and dark phases, respectively.** (B)** Locomotor activities monitored over 3 weeks (1 week under a 12 h light/dark cycle and 2 weeks under constant darkness) for *ob/ob*, HFD and control mice. ZT, Zeitgeber time. **(C)** Locomotor activities monitored over 1 week for *ob/ob*, HFD and control mice kept under a 12 h light/dark condition. Data are mean ± SD (*n* = 7). *p < 0.05 (one-way ANOVA and Bonferroni post hoc test).** (D)** Percent of total activity for the light period. Data are mean ± SD (*n* = 7). *p < 0.05 (one-way ANOVA and Bonferroni post hoc test).** (E)** Energy expenditure (EE) monitored over 3 days for *ob/ob*, HFD and control mice kept under a 12 h light/dark condition. Data are mean ± SD (*n* = 3). *p < 0.05 (one-way ANOVA and Bonferroni post hoc test). **(F)** Percent of total EE for the light period. Data are mean ± SD (*n* = 3). *p < 0.05 (one-way ANOVA and Bonferroni post hoc test). **(G)** Percent of total food intake for the light period. Data are mean ± SD (*n* = 3). *p < 0.05 (one-way ANOVA and Bonferroni post hoc test).

Because adipocyte clock has a role in regulating feeding rhythms and energy balance 20, we tested whether obesity affects circadian rhythms related to energy metabolism. Both *ob/ob* and HFD-induced obese mice showed a decreased locomotor activity with no significant change in circadian period based on actogram analysis (Figure 2A-B & Figure S4). The decreases in daily activities were attributed to reductions in nighttime activities as the daytime activities were increased (Figure 2C). Thus, circadian rhythm in locomotor activity was attenuated in obese mice with an increased percent of activity in the daytime (Figure 2D). The obese mice also had decreased daily energy expenditure (EE) compared to control mice (Figure 2E), consistent with the changes in locomotor activity. Likewise, the EE rhythm was dampened with an increased percent of EE in the light period (Figure 2F). Consistently, circadian rhythm in the daily food intake was also attenuated in obese mice with an increased percent of food intake in the daytime (Figure 2G). Intriguingly, the obesity-associated circadian phenotypes were very similar to those of mice with *Bmal1*-specifically deleted in the adipocytes 20. Altogether, obesity in mice attenuates circadian rhythms in locomotor activity, EE, and feeding behavior, that may be linked to dysfunction of adipocyte clock.

### Obesity downregulates glutamine and methionine in mouse WAT

**Figure 3:**
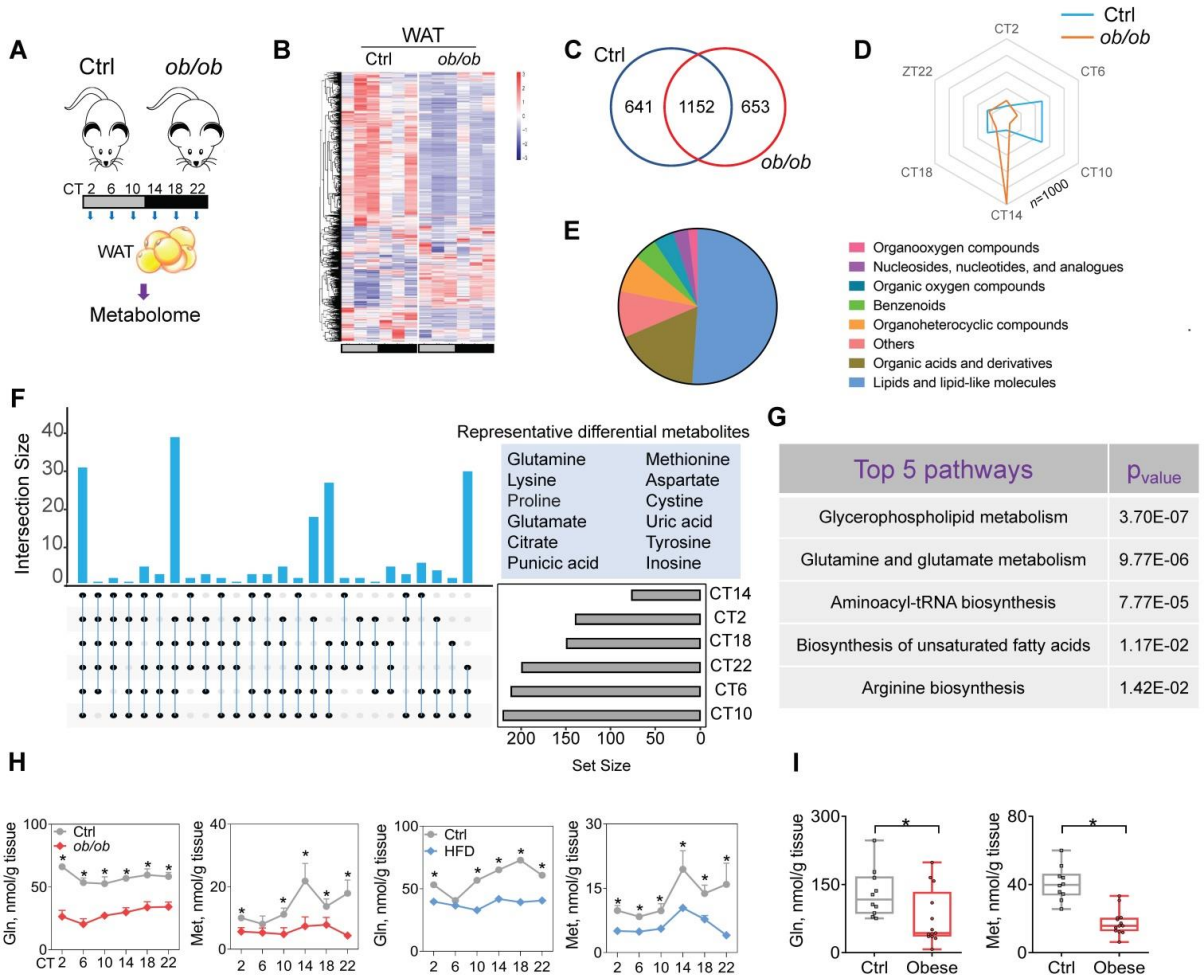
** Obesity downregulates glutamine and methionine in mouse WAT. (A)** Schematic diagram for metabolomic analysis. **(B)** Heatmap for oscillating metabolites in WAT of *ob/ob* and control mice at six circadian time points. Red indicates high expression, and blue indicates low expression of metabolites as shown in the scale bar.** (C)** Venn diagram showing numbers of oscillating metabolites in WAT of *ob/ob* and control mice. **(D)** Radar diagram showing phase distribution of metabolites in WAT of *ob/ob* and control mice.** (E)** Pie diagram showing percentages of oscillating metabolites in WAT of *ob/ob* and control mice. **(F)** Upset plot showing differential metabolites (representative ones are shown in right panel) occurring at more than two circadian times simultaneously. **(G)** KEGG analysis of obesity-associated differential metabolites in WAT (Top 5 pathways are shown). **(H)** Glutamine and methionine levels in WAT of *ob/ob* and control mice at 6 circadian time points. Data are mean ± SD (*n* = 5). *p < 0.05 (two-way ANOVA and Bonferroni post hoc test).** (I)** Glutamine and methionine levels in omental WAT of obese and lean humans. Data are mean ± SD (*n* = 13 for obese & n=10 for lean). *p < 0.05 (t-test).

Because metabolites (particularly oscillating metabolites such as melatonin, glucose, heme and bilirubin) are potential modulators of circadian clock and obesity is associated with metabolic changes in WAT 38-39,40,41, we investigated a role of metabolites in regulation of adipocyte clock by obesity. To this end, we performed global 24-hr metabolomics profiling of WAT from *ob/ob* and normal mice (Figure 3A). *ob/ob* mice showed considerable changes in a large number of metabolites in WAT (Figure 3B & Supplementary excel file). JTK_CYCLE algorithm identified 1793 oscillating metabolites in normal mice, and > 35% of them were no longer rhythmic in obese mice (Figure 3C). Analysis of the oscillating metabolites revealed a significantly altered phase distribution in *ob/ob* mice (Figure 3D). Differential WAT metabolites (based on metaX software calculation) between *ob/ob* and normal mice were observed at one or more of six circadian times. A majority of differential metabolites were lipids and organic acids (mainly including amino acids) (Figure 3E). We further examined differential metabolites occurring at more than two circadian times simultaneously, which were considered as the ones altered the most (Figure 3F). These metabolites included glutamine, methionine, glutamate and citrate (Figure 3F). KEGG enrichment analysis of differential metabolites identified several specific pathways deregulated in obese mice, including glycerophospholipid metabolism, glutamine and glutamate metabolism, and aminoacyl-tRNA biosynthesis (Figure 3G). Glycerophospholipid mainly serves as a structural component of biological membranes 42, and its role in regulating circadian clock may be excluded. Mass spectrometric analyses demonstrated downregulation of glutamine and glutamate (a metabolic product of glutamine) in* ob/ob* mice and loss of their circadian rhythms, consistent with the KEGG findings (Figure 3H & Supplementary excel file). Furthermore, we examined the amino acids (key players) involved in the pathway of aminoacyl-tRNA biosynthesis, and found that glutamine, glutamate and methionine were downregulated to the largest degree (Figure 3H & Supplementary excel file). Supporting the mouse data, both glutamine and methionine were decreased in omental WAT of obese patients as compared to lean individuals (Figure 3I). Glutamine and methionine have been shown to regulate gene transcription and expression 43-44,45, thus may be potential modulators of circadian clock. Altogether, obesity reduces glutamine and methionine, and disrupts their circadian rhythms in mouse WAT.

### Obesity impacts the *Bmal1* epigenetics in a glutamine- and methionine-dependent manner

**Figure 4:**
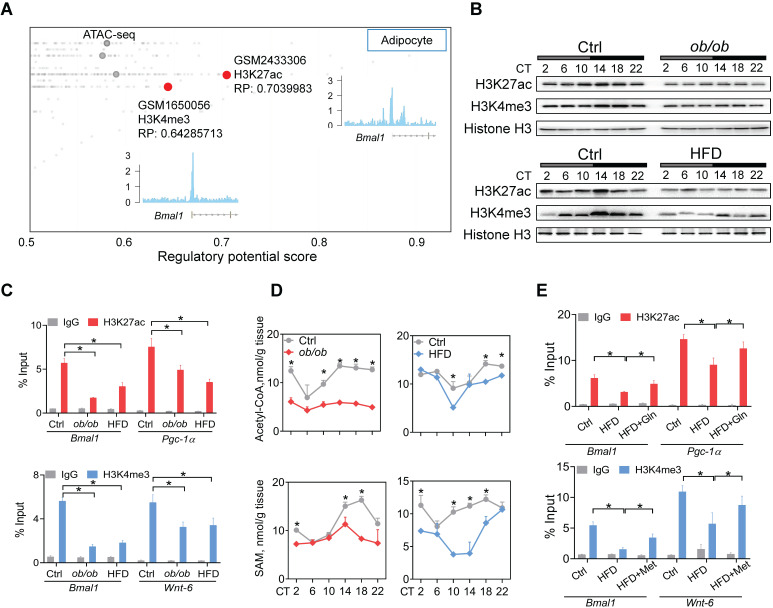
** Obesity impacts epigenetics of *Bmal1* in a glutamine- and methionine-dependent manner. (A)** Identification of H3K27ac and H3K4me3 as histone marks of *Bmal1* gene in adipocytes according to Cistrome database (accession numbers: GSM2433306 and GSM1650056). All Cistrome data have been carefully curated and processed with a streamlined analysis pipeline and evaluated with comprehensive quality control metrics^46^. **(B)** Protein expression of H3K27ac and H3K4me3 in WAT of *ob/ob* (upper panel), HFD (lower panel) and control mice **(C)** Enrichments of H3K27ac and H3K4me3 proteins at *Bmal1* promoter in WAT of *ob/ob*, HFD and control mice at CT6. *Pgc-1α* and *Wnt-6* were used as positive controls for H3K27ac and H3K4me3, respectively. Data are mean ± SD (*n* = 3). *p < 0.05 (two-way ANOVA and Bonferroni post hoc test). **(D)** Levels of acetyl-CoA and S-adenosyl methionine (SAM) in WAT of *ob/ob*, HFD and control mice at 6 circadian time points. Data are mean ± SD (*n* = 5). *p < 0.05 at individual time points as determined by two-way ANOVA and Bonferroni post hoc test. **(E)** ChIP assays showing enrichments of H3K27ac and H3K4me3 at *Bmal1* promoter in WAT of HFD and control mice administered with glutamine, methionine or vehicle. Data are mean ± SD (*n* = 3). *p < 0.05 as determined by two-way ANOVA and Bonferroni post hoc test.

Metabolism of glutamine in the citric acid cycle generates citrate and contributes to production of acetyl-CoA, the acetyl carrier for histone acetylation 47. Methionine is an essential metabolite for biosynthesis of SAM which functions as a substrate for histone methylation 46. Recent studies have proposed glutamine and methionine as critical regulators of gene transcription via epigenetic modifications 48. We thus investigated whether deregulation of glutamine and methionine in obese mice impacts epigenetic modifications of clock genes. According to Cistrome database 49, H3K27ac and H3K4me3 (both are associated with active transcription) were histone marks highly enriched at *Bmal1* promoter in adipocytes (Figure 4A). Both *ob/ob* and HFD-induced obese mice had lower levels of total H3K27ac and H3K4me3 proteins in WAT as compared to control mice (Figure 4B). However, other histone marks such as H3K9ac, H3K9me2, H3K9me3 and H3K27me3 were unaffected (Figure S5). ChIP assays showed significantly reduced enrichments of H3K27ac and H3K4me3 at the *Bmal1, Pgc-1α* and *Wnt-6* promoters in WAT of obese mice (Figure 4C). Furthermore, we observed lower levels of acetyl-CoA and SAM in obese WAT (Figure 4D). This was accompanied by reductions in glutamate and citrate (Supplementary excel file). Moreover, glutamine or methionine administration to obese mice (HFD-induced) partially rescued epigenetic changes in *Bmal1, Pgc-1α* and *Wnt-6* (Figure 4E). Additionally, C646 (a histone acetyltransferase inhibitor 50) and MM-102 (an H3K4 histone methyltransferase inhibitor 51) dose-dependently attenuated the effects of glutamine and methionine on *Bmal1* expression as they reduced the level of H3K27ac or H3K4me3 in 3T3-L1 adipocytes (Figure S6). Taken together, these findings indicate that deregulation of glutamine and methionine in obese mice reduces H3K27ac and H3K4me3 at *Bmal1* promoter, thereby potentially downregulating *Bmal1* transcription and expression.

### Glutamine and methionine enhance BMAL1 expression in WAT and protect mice against developing obesity

**Figure 5:**
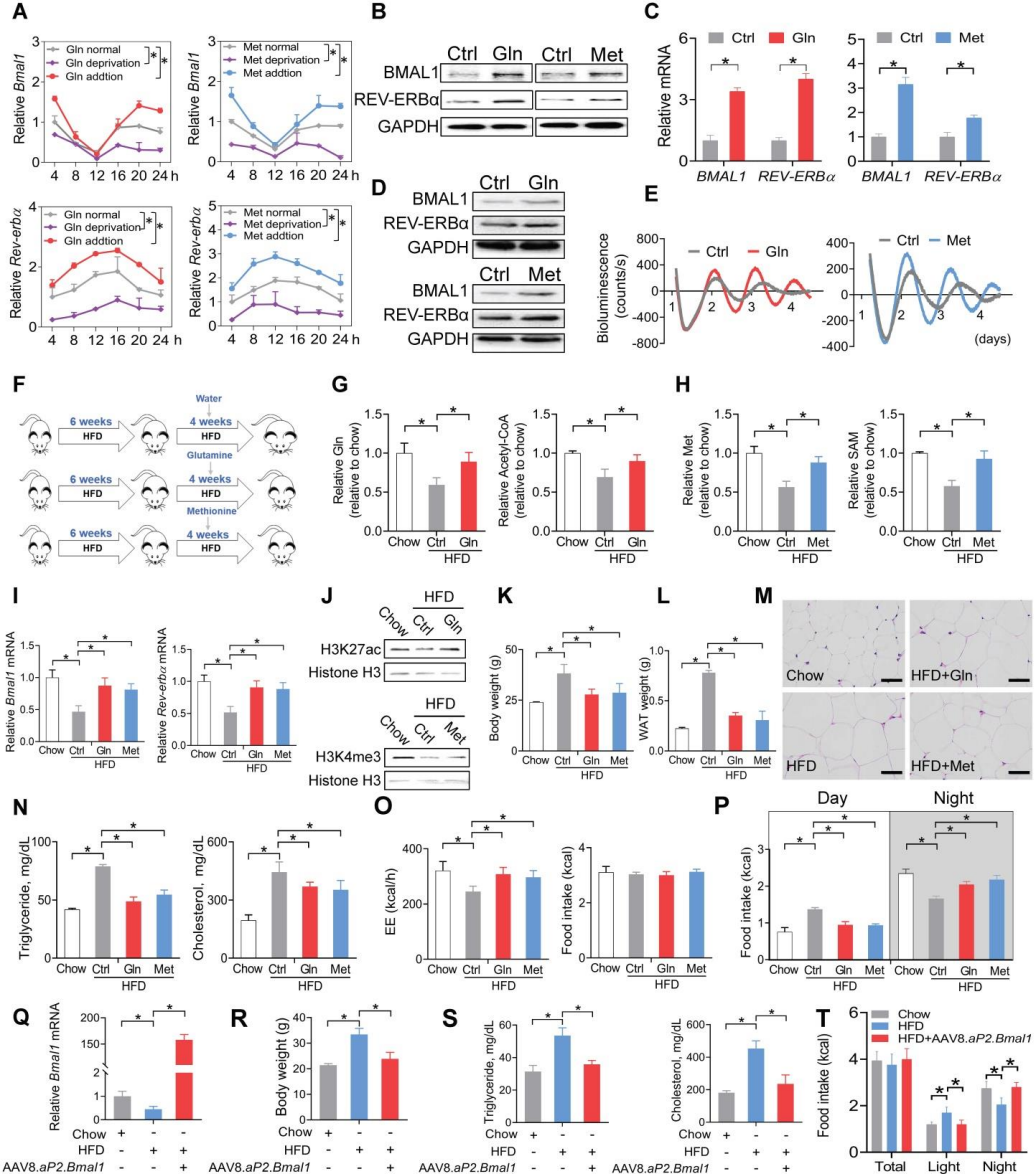
** Glutamine and methionine enhance BMAL1 expression in WAT and protect mice against developing obesity. (A)** Effects of glutamine/methionine addition or deprivation on mRNAs of *Bmal1* and* Rev-erbα* in synchronized 3T3-L1 adipocytes. Data are mean ± SD (*n* = 3). *p < 0.05 as determined by two-way ANOVA and Bonferroni post hoc test.** (B)** Protein expression of BMAL1 and REV-ERBα in 3T3-L1 adipocytes treated with glutamine or methionine. **(C)** mRNA expression of BMAL1 and REV-ERBα in human adipocytes treated with glutamine or methionine. Data are mean ± SD (*n* = 3). *p < 0.05 (t-test).** (D)** Protein expression of BMAL1 and REV-ERBα in human adipocytes treated with glutamine or methionine. **(E)** Bioluminescent recordings of *BMAL1-Luc* U2OS cells after glutamine or methionine treatment. **(F)** Experimental protocol for assessment of glutamine and methionine on adipocyte clock and obesity development. **(G)** Glutamine and acetyl-CoA levels in WAT derived from HFD- and chow-fed mice at CT6. Data are mean ± SD (*n* = 5). *p < 0.05 (one-way ANOVA and Bonferroni post hoc test). **(H)** Methionine and SAM in WAT derived from HFD- and chow-fed mice at CT6. Data are mean ± SD (*n* = 5). *p < 0.05 (one-way ANOVA and Bonferroni post hoc test). **(I)** mRNA expression of *Bmal1* and *Rev-erbα* in WAT derived from HFD- and chow-fed mice at CT6. Data are mean ± SD (*n* = 5). *p < 0.05 (one-way ANOVA and Bonferroni post hoc test). **(J)** Protein expression of H3K27ac and H3K4me3 in WAT derived from HFD- and chow-fed mice at CT6.** (K)** Body weight of HFD- and chow-fed mice. Data are mean ± SD (*n* = 5). *p < 0.05 (one-way ANOVA and Bonferroni post hoc test). **(L)** WAT weight of HFD- and chow-fed mice at CT6. Data are mean ± SD (*n* = 5). *p < 0.05 (one-way ANOVA and Bonferroni post hoc test).** (M)** H&E staining of WAT derived from HFD- and chow-fed mice. Scale bar = 50 μm. **(N)** Serum triglycerides and cholesterol in HFD and chow mice at CT6. Data are mean ± SD (*n* = 5). *p < 0.05 (one-way ANOVA and Bonferroni post hoc test). **(O)** EE (left panel) and food intake (right panel) monitored over 3 days for HFD- and chow-fed mice kept under a 12 h light/dark condition. Data are mean ± SD (*n* = 5). *p < 0.05 (one-way ANOVA and Bonferroni post hoc test).** (P)** Food intake (day and night) was monitored over 3 days for HFD- and chow-fed mice kept under a 12 h light/dark condition. **(Q)** Effects of AAV8.*aP2*.*Bmal1* on *Bmal1* mRNA in WAT in obese mice induced by HFD.** (R)** Effects of AAV8.*aP2*.*Bmal1* on body weight of obese mice induced by HFD.** (S)** Effects of AAV8.*aP2*.*Bmal1* on serum triglycerides and cholesterol in obese mice induced by HFD. **(T)** Effects of AAV8.*aP2*.*Bmal1* on food intake monitored over 3 days for obese mice kept under a 12 h light/dark condition. Data are mean ± SD (*n* = 5). *p < 0.05 (one-way ANOVA and Bonferroni post hoc test).

We next tested whether glutamine and methionine can regulate expression of* Bmal1* and related clock genes. Addition of glutamine or methionine enhanced the mRNA expression of *Bmal1* and its target clock gene (i.e., *Rev-erbα*) in synchronized 3T3-L1 adipocytes, whereas deprivation of glutamine or methionine reduced their expression levels (Figure 5A). Consistently, BMAL1 and REV-ERBα proteins in 3T3-L1 adipocytes were increased by the two amino acids (Figure 5B). Likewise, mRNA and protein levels of both BMAL1 and REV-ERBα were elevated in human visceral adipocytes treated with glutamine or methionine (Figure 5C-D). We performed real-time bioluminescence recordings of a *BMAL1*-luciferase reporter in U2OS cells treated with glutamine or methionine or vehicle (Figure 5E). Glutamine and methionine increased the *BMAL1*-luciferase reporter activity, suggestive of their abilities to enhance *Bmal1* transcription (Figure 5E). We further examined the *in vivo* effects of glutamine and methionine on adipocyte clock. We initiated the study with 10-week-old mice that had been maintained on a HFD for 6 weeks (Figure 5F). The mice continued on HFD and were treated with glutamine or methionine in drinking water (Figure 5F). We confirmed that glutamine and methionine treatments did increase the concentrations of glutamine/acetyl-CoA and methionine/SAM in mouse WAT, respectively (Figure 5G-H). Glutamine- and methionine-treated mice showed higher adipocyte expression levels of *Bmal1* and* Rev-erbα* as well as increased H3K27ac and H3K4me3 as compared to vehicle-treated mice (Figure 5I-J). Further, glutamine and methionine significantly decreased the body weight, and reduced the mass of WAT and the size of adipocytes, indicating amelioration of mouse obesity (Figure 5K-M). They also protected mice against developing obesity-associated metabolic complications such as hyperlipidemia and hypercholesterolemia (Figure 5N). To examine whether restoration of clock genes is an effect of glutamine and methionine rather than weight loss, we initiated short-term (two weeks) treatment experiments with chow- and HFD-fed mice. Short-term treatment with methionine or glutamine did not impact animal weight, but enhanced the expression of clock genes in WAT, however, obesity-associated metabolic parameters (such as serum triglycerides and cholesterol) were unaffected (Figure S7). Thus, methionine and glutamine had casual effects on adipocyte clock. As the feeding rhythm and EE were normalized in glutamine- and methionine-treated obese mice (Figure 5O-P), we wondered whether *Bmal1* enhancement is linked to obesity protection. To test this, we overexpressed *Bmal1* in adipocytes in HFD-fed obese mice using recombinant adeno-associated virus serotype 8 (AAV8) vector encoding* Bmal1* driven by an adipocyte-specific *aP2* promoter (named “AAV8.*aP2.Bmal1”*), and examined its effects on obesity and associated comorbidities (Figure 5Q-T). We found that adipocyte-specific overexpression of *Bmal1* attenuated obesity and metabolic abnormalities (Figure 5R-T). This is consistent with a prior study by Paschos *et al* in which *Bmal1* malfunction in adipocytes results in obesity 20. Altogether, these findings suggest that glutamine and methionine can restore the function of adipocyte clock to ameliorate obesity in mice.

### SLC1A5, a dual glutamine and methionine uptake transporter, is downregulated in mouse WAT

**Figure 6:**
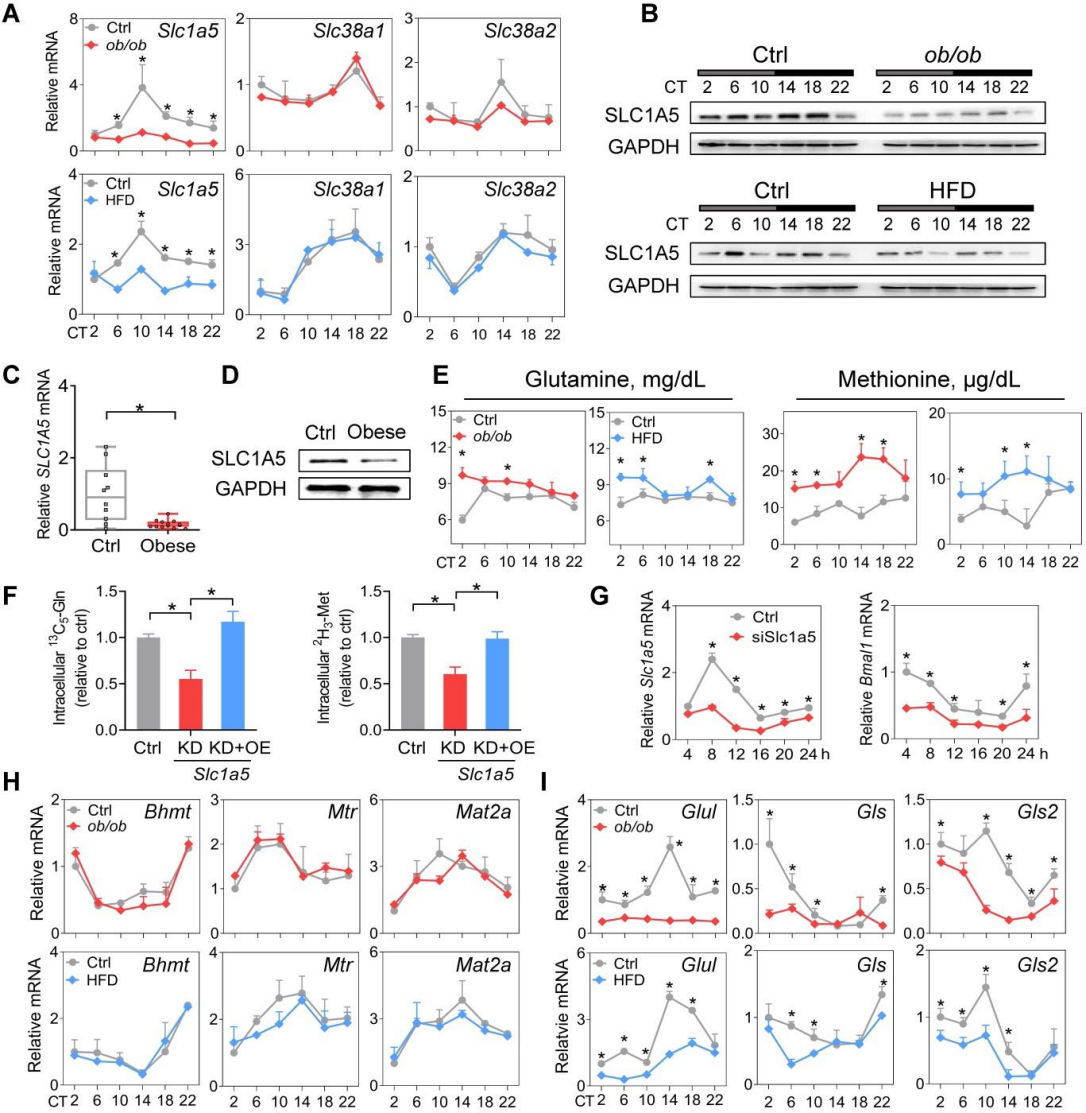
** SLC1A5, a dual glutamine and methionine uptake transporter, is downregulated in mouse WAT. (A)** mRNA expression of three dual glutamine and methionine uptake transporters (*Slc1a5*, *Slc38a1* and *Slc38a2*) in WAT of *ob/ob*, HFD and control mice at 6 circadian times. Data are mean ± SD (*n* = 5). *p < 0.05 at individual time points as determined by two-way ANOVA and Bonferroni post hoc test. **(B)** Protein expression of SLC1A5 in WAT of *ob/ob* (top panel), HFD (bottom panel) and control mice at 6 circadian times. **(C and D)** mRNA **(C)** and protein **(D)** expression of SLC1A5 in the omental fat of obese or lean humans. Data are mean ± SD (*n* = 13 for obese & *n* = 10 for lean). *p < 0.05 (t-test). **(E)** Serum glutamine and methionine levels in *ob/ob*, HFD and control mice at 6 circadian times. Data are mean ± SD (*n* = 5). *p < 0.05 at individual time points as determined by two-way ANOVA and Bonferroni post hoc test.** (F)** Uptake of [^13^C_5_]-glutamine and [^2^H_3_]-methionine in 3T3-L1 adipocytes transfected with siSlc1a5 (for gene knockdown/KD) or control with or without *Slc1a5* overexpression (OE). Data are mean ± SD (*n* = 3). *p < 0.05 (one-way ANOVA and Bonferroni post hoc test)**. (G)** *Slc1a5* and *Bmal1* mRNAs in synchronized 3T3-L1 adipocytes transfected with siSlc1a5 or control. *p < 0.05 at individual time points as determined by two-way ANOVA and Bonferroni post hoc test.** (H)** mRNA expression of methionine-related enzymes (*Bhmt*, *Mtr* and *Mat2a*) in WAT derived from *ob/ob*, HFD and control mice. Data are mean ± SD (*n* = 5). **(I)** mRNA expression of glutamine-related enzymes (*Glul*, *Gls* and *Gls2*) in WAT derived from *ob/ob*, HFD and control mice. Data are mean ± SD (*n* = 5). *p < 0.05 at individual time points as determined by two-way ANOVA and Bonferroni post hoc test.

Amino acid levels in the body are tightly regulated by transport and metabolic processes 52. SLC1A5, SLC38A1 and SLC38A2 are three critical transporters responsible for cellular uptake of both glutamine and methionine 53. SLC1A5 expression was considerably downregulated, whereas the expression levels of *Slc38a1* and *Slc38a2* were unaffected in WAT in obese mice compared to normal mice (Figure 6A-B). Consistently, we observed a lower level of SLC1A5 in omental WAT of obese patients (Figure 6C-D). Downregulation of SLC1A5 may account for reduced glutamine and methionine in WAT of obese mice. Supporting this, circulating glutamine and methionine levels were increased in obese mice (Figure 6E). Furthermore, S*lc1a5* knockdown in 3T3-L1 adipocytes resulted in reduced uptake of both [^13^C_5_]-glutamine and [^2^H_3_]-methionine, however, overexpression of *Slc1a5* reversed these changes (Figure 6F). Moreover, knockdown of *Slc1a5* led to decreased *Bmal1* expression and disrupted rhythm in synchronized 3T3-L1 adipocytes (Figure 6G). We analyzed the WAT expression of glutamine- and methionine-related enzymes in obese and control mice. Obesity did not affect the expression of *Bhmt*, *Mtr* and *Mat2a* (Figure 6H). However,* Gls* and *Gls2* (glutaminases, responsible for metabolism of glutamine to glutamate) were downregulated (Figure 6I). We also observed reduced expression of *Glul* (glutamine synthetase, catalyzing the formation of glutamine from glutamate) in obese mice (Figure 6I), consistent with a prior study 45. We argued that reduced glutamine and methionine pools in WAT were mainly attributed to impaired uptake transport mediated by SLC1A5. Contributions of the reductions in the three enzymes GLUL, GLS and GLS2 (catalyzing interconversion between glutamine and glutamate, a futile recycling) to a reduced glutamine pool may be negligible because of concurrent reductions in both glutamine and glutamate in obese WAT (Figure 3H & Supplementary excel file).

### Impaired PPAR-γ in mouse WAT reduces SLC1A5 expression via a transcriptional mechanism

**Figure 7:**
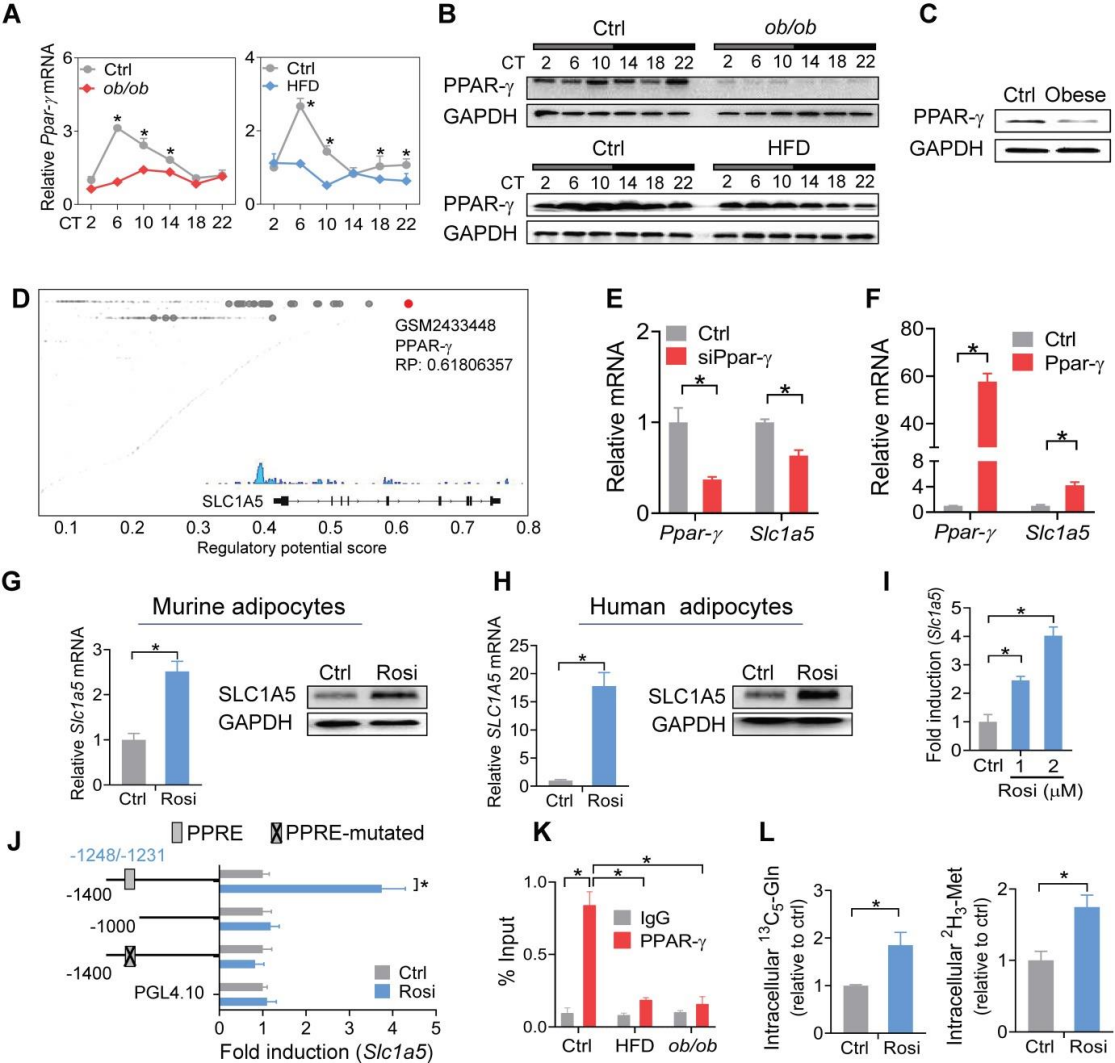
** Impaired PPAR-γ in mouse WAT reduces SLC1A5 expression via a transcriptional mechanism. (A and B)** PPAR-γ mRNA **(A)** and protein **(B)** in WAT derived from *ob/ob*, HFD and control mice at 6 circadian times. Data are mean ± SD (*n* = 5). *p < 0.05 at individual time points as determined by two-way ANOVA and Bonferroni post hoc test. **(C)** PPAR-γ protein in the omental fat of obese and lean subjects. **(D)** Identification of PPAR-γ as a potential regulator of *Slc1a5* gene in adipocytes according to Cistrome database (accession number: GSM2433448). All Cistrome data have been carefully curated and processed with a streamlined analysis pipeline and evaluated with comprehensive quality control metrics 46. **(E)** Effects of *Ppar-γ* knockdown (after 3T3-L1 cells differentiation) on *Slc1a5* mRNA in 3T3-L1 adipocytes. Data are mean ± SD (*n* = 3). *p < 0.05 (t-test).** (F)** Effects of *Ppar-γ* overexpression on *Slc1a5* mRNA in 3T3-L1 adipocytes. Data are mean ± SD (*n* = 3). *p < 0.05 (t-test).** (G)** SLC1A5 mRNA and protein in 3T3-L1 adipocytes treated with rosiglitazone (Rosi, a PPAR-γ agonist). Data are mean ± SD (*n* = 3). *p < 0.05 (t-test). **(H)** SLC1A5 mRNA and protein in human adipocytes treated with rosiglitazone. Data are mean ± SD (*n* = 3). *p < 0.05 (t-test). **(I)** Effects of rosiglitazone (1 and 2 μM) on the activities of *Slc1a5-Luc* reporter (-1400/+100 bp) in 3T3-L1 adipocytes. Data are mean ± SD (*n* = 6). *p < 0.05 (one-way ANOVA and Bonferroni post hoc test). **(J)** Effects of rosiglitazone (1 μM) on the activities of various versions of *Slc1a5-Luc* reporters (-1400/+100 bp, -1000/+100 bp and a PPRE-mutated version) in 3T3-L1 adipocytes. Data are mean ± SD (*n* = 6). *p < 0.05 (t-test). **(K)** ChIP assays showing recruitment of PPAR-γ protein to the PPRE element of *Slc1a5* promoter in WAT of *ob/ob*, HFD and control mice at CT6. Data are mean ± SD (*n* = 3). *p < 0.05 at individual time points as determined by two-way ANOVA and Bonferroni post hoc test.** (L)** Effects of rosiglitazone on [^13^C_5_]-glutamine and [^2^H_3_]-methionine uptake into 3T3-L1 adipocytes. Data are mean ± SD (*n* = 3). *p < 0.05 (t-test).

PPAR-γ is a critical nuclear receptor in obesity development and downregulated in obese conditions 54. Obesity-induced downregulation of PPAR-γ is likely attributed to induction of the proinflammatory cytokine TNF-a, a repressor of PPAR-γ 55. We confirmed that PPAR-γ expression was reduced in WAT of both mice and humans with obesity (Figure 7A-C). Also, PPAR-γ was rhythmically expressed in normal mice, but its rhythm was disrupted in obese mice (Figure 7A-B). We thus wondered whether PPAR-γ plays a role in obesity-repression of SLC1A5. ChIP-sequencing revealed PPAR-γ as a potential transcriptional regulator of SLC1A5 in adipose tissue (Figure 7D). We found knockdown of *Ppar-γ* decreased, whereas overexpression of *Ppar-γ* increased, the mRNA level of *Slc1a5* in 3T3-L1 adipocytes (Figure 7E-F). Consistently, activation of PPAR-γ by rosiglitazone (a specific agonist) increased the mRNA and protein of SLC1A5 in 3T3-L1 adipocytes (Figure 7G). Rosiglitazone can also induce SLC1A5 expression in human adipocytes (Figure 7H). Furthermore, rosiglitazone induced the luciferase activity driven by a -1.4 kb *Slc1a5* promoter in a dose-dependent manner (Figure 7I). These data indicated that PPAR-γ transcriptionally regulated the expression of SLC1A5. Moreover, truncation and mutation analyses identified a PPRE element (i.e., -1248/-1231 bp) in *Slc1a5* promoter responsible for PPAR-γ action (Figure 7J). ChIP assays confirmed recruitment of WAT PPAR-γ to the PPRE element of *Slc1a5* in normal mice (Figure 7K), however, such recruitment was reduced in obese mice (Figure 7K). In addition, PPAR-γ activation enhanced the uptake of [^13^C_5_]-glutamine and [^2^H_3_]-methionine into 3T3-L1 adipocytes (Figure 7L), accompanied by enhanced enrichments of both H3K27ac and H3K4me3, and increased *Bmal1* expression (Figure S8). However, the effect of PPAR-γ activation on *Bmal1* expression was attenuated by *Slc1a5* knockdown (Figure S9). Taken together, PPAR-γ is down-regulated in obese WAT and is a direct transcriptional activator of *Slc1a5* gene. Thereby, impaired PPAR-γ in WAT of obese mice causes reductions in SLC1A5 expression and in adipocyte uptake of both glutamine and methionine.

## Discussion

We have observed perturbance of adipocyte clock (a peripheral circadian clock) in obese conditions. Importantly, we have revealed PPAR-γ as an integrator of obesity and adipocyte clock. Obesity causes a reduction in PPAR-γ, which provokes downregulation of SLC1A5 (a direct target of PPAR-γ and a dual uptake transporter of glutamine and methionine) and reductions in adipocyte glutamine and methionine (two epigenetic activators of *Bmal1*), leading to disruption of *Bmal1* and other clock genes and thus to impaired adipocyte clock function. The mechanisms for obesity-repression of PPAR-γ have been established in the literature. Obese adipose tissue releases the proinflammatory cytokine TNF-α which inhibits PPAR-γ expression via suppressing C/EBPδ (a transcriptional activator of *PPAR-γ* gene) and enhancing HDAC3 (a PPAR-γ corepressor) activity in the nucleus 54-57. Additional mechanism involves free fatty acids elevated in obese adipose tissue 58,59. Free fatty acids inhibit PPAR-γ expression in a TLR4/ER stress-dependent manner 60. In a prior study, adipocyte-specific deletion of *Bmal1* results in obesity in mice due to an attenuated rhythm in food intake (i.e., increased food intake in daytime) caused by temporal changes in circulating polyunsaturated fatty acids and in expression of neurotransmitters for appetite regulation 20. Therefore, we propose a vicious cycle between circadian disruption and obesity development (Figure S10), in which obesity can be both a cause and an effect of circadian disruption, and vice versa. This vicious cycle appears to perpetuate and intensify obesity, providing an explanation for the increasing prevalence of obesity worldwide.

Clock genes such as* Rev-erbα/β*, *Per1/2*, *Cry1/2*, *Rorα* and* Dbp* were also disrupted in obese adipose tissue in addition to *Bmal1* (Figure 1 & Figure S2). It is most likely that downregulation of these clock genes is secondary to the change in BMAL1 expression as the transcription of these genes is directly driven by BMAL1 and they are BMAL1 target genes 36,37. It is thus proposed that BMAL1 is a fundamental factor linking obesity to circadian clock (adipocyte clock). In addition to disrupted clock gene expression, obesity results in disturbance of feeding rhythm in mice (Figures 1 & 2), that phenocopies adipocyte-specific knockout of *Bmal1* 20. Therefore, disturbed feeding rhythm in obesity is mainly attributed to impaired expression of adipocyte *Bmal1*, which has an effect on hypothalamic feeding centers via regulating circulating and hypothalamic polyunsaturated fatty acids 20. It is noteworthy that obesity is also associated with decreased locomotor activity in mice (Figure 2). However, SCN clock genes were unaffected in obese mice, consistent with prior studies 27,61,62. We speculate that reduced locomotion in obesity is regulated by the nervous systems outside the SCN (e.g., the dopamine and endocannabinoid systems) 63-64,65.

Both PPAR-γ mRNA and protein oscillate with circadian time in adipose tissue in normal mice, however, the protein is phase-delayed about 4 hours compared to the mRNA (Figure 7A-B). This is probably due to a time lag in the translation from mRNA to protein, as noted previously for hepatic PPAR-γ 66. The PPAR-γ protein is in phase with the *Slc1a5* mRNA, supporting PPAR-γ as a transcriptional activator of *Slc1a5* and as a main driver of *Slc1a5* rhythm (Figures 6A & 7B). Although the mechanism for circadian oscillations in PPAR-γ remains elusive, nocturnin (*Noc*, a circadian deadenylase and a post-transcriptional modifier of mRNAs) may regulate the circadian expression pattern of PPAR-γ as *Noc*-deficient mice show attenuated diurnal rhythm in *Ppar-γ* mRNA 67. We analyzed the expression of *Noc* in obese adipose tissue, and its level was unaltered (Figure S11). Therefore, the blunting of PPAR-γ rhythm in obese adipose tissue is most likely attributed to circadian inhibiting effects of TNF-α, whose level varies with the times of the day 56,68.

We have shown a reduced glutamine level in obese adipose tissue consistent with a recent report by Petrus *et al* 45. Reduction in adipocyte glutamine is mainly attributed to impaired expression of SLC1A5, which mediates cellular uptake of glutamine. By contrast, Petrus *et al* ascribed the reduction of glutamine to the lowered level of GLUL (glutamine synthetase), an enzyme generates glutamine by using glutamate as a substrate. However, glutamine and glutamate are interchangeable, and GLS (glutaminase, also reduced in obese adipose tissue) catalyzes the formation of glutamate from glutamine 69. If glutamine is decreased due to reduced catabolism of glutamate, one may expect to see an elevation in adipocyte glutamate. This contradicts with the observation that glutamate is considerably reduced in obese adipose tissue (Supplementary excel file). Therefore, we argue that impaired uptake of glutamine from the blood circulation to adipocytes accounts for the reductions in both glutamine and glutamate. Interestingly, glutamine treatment can ameliorate obesity and related comorbidities in mice (Figure 5), consistent with previous observations that glutamine supplementation reduces obesity with an improvement in systemic insulin action 70,71. Likewise, methionine protects mice against developing obesity and related comorbidities. The main mechanism of their actions refers to the restoration of adipocyte clock which regulates energy balance (feeding rhythm) and obesity development (Figure 5). Since clock genes such as *Rev-erbα* act as inflammatory repressors 72, there is a possibility that clock restoration reduces adipocyte inflammation and contributes to the protecting effects of glutamine and methionine on obesity (a condition of chronic low-grade inflammation).

We focused on analysis of changes in clock genes in obese models because we intended to clarify the mechanisms by which obesity regulates circadian clock. We have revealed that obesity causes considerable disruption of clock genes in WAT. It is noteworthy that affected genes should not be limited to clock genes considering that H3K27ac and H3K4me3 are histone marks broadly distributed at active promoters in the genome. In fact, we found that non-clock genes such as *Pgc-1α* and* Wnt-6* (with known H3K27ac or H3K4me3 distribution) are also downregulated in WAT of HFD-fed mice, accompanied by reduced enrichment of H3K27ac or H3K4me3 at the gene promoters (Figures S12, 4C). We have shown that H3K27ac and H3K4me3 are altered in response to the reductions of glutamine and methionine in obese models, while other histone marks potentially affecting the transcription of clock genes are unaffected (Figure S5). The specific effect of methionine change on H3K4me3 is consistent with prior studies in which methionine metabolism and the SAM level affect the expression of H3K4me3, while having no influences on other histone methylation marks such as H3K4me1, H3K4me2, H3K9me2, H3K9me3, H3K27me3, H3K36me3 and H3K79me3 73-74,75,76. However, the mechanism by which the methionine effect is specific to H3K4me3 remains unknown. Likewise, why the glutamine effect is specific to H3K27ac is unaddressed.

HFD is known to affect circadian gene expression in the tissues, particularly the liver 77-78,79. To examine the impact of HFD-induced obesity (an obese condition) on circadian clock, we attempted to minimize the effects of HFD by feeding obese mice (that had been maintained on HFD for 10 weeks) and controls a normal chow diet for one week prior to gene expression analysis. Therefore, the obese condition rather than HFD is the direct cause of disturbances in adipocyte clock and circadian behaviors (Figures 1 & 2). Supporting this, mice fed an HFD for 3 days (in the absence of obesity) showed unaffected expression of clock genes in adipose tissue (Figure S13). In addition, *ob/ob* mice maintained on a normal chow diet developed obesity and showed the circadian phenotypes similar to those of HFD-induced obese mice (Figures 1 & 2), emphasizing an association of adipocyte cock with obese conditions but not with diets.

In conclusion, obesity disrupts adipocyte clock in mice. Mechanistically, obesity causes a reduction in PPAR-γ, which provokes downregulation of SLC1A5 (a direct target of PPAR-γ and a dual uptake transporter of glutamine and methionine) and reductions in adipocyte glutamine and methionine (two epigenetic activators of *Bmal1*), contributing to disruption of *Bmal1* and other clock genes and thus to impaired adipocyte clock. Therefore, PPAR-γ integrates obesity and adipocyte clock, facilitating a vicious cycle between circadian disruption and obesity development.

## Methods

### Materials

Anti-BMAL1 (ab3350), anti-H3K27ac (ab4729), anti-H3K4me3 (ab8580), anti-H3K9me3 (ab8898) and anti-GAPDH (ab8245) antibodies were purchased from Abcam (Cambridge, UK). Anti-REV-ERBα (14506-1-AP), anti-DBP (12662-1-AP), anti-SLC1A5 (20350-1-AP), and anti-Histone H3 (17168-1-AP) antibodies were purchased from Proteintech (Wuhan, China). Anti-PPAR-γ (2443), anti-H3K9ac (9649), anti-H3K9me2 (4658), anti-H3K27me3 (9733) and anti-rabbit IgG (2729) were purchased from Cell Signaling Technology (Danvers, MA). Glutamine, methionine, C646 and MM-102 were purchased from Tsbiochem (Shanghai, China). [^13^C_5_]-glutamine and [^2^H_3_]-methionine were purchased from Zzbio (Shanghai, China). Dexamethasone was purchased from Sigma-Aldrich (St. Louis, MO). RNAiso Plus reagent, PrimeScript RT Master Mix and ChamQ Universal SYBR qPCR Master Mix were purchased from Vazyme (Nanjing, China). Beetle luciferin was purchased from Promega (Madison, WI). siRNAs for *Slc1a5* and *Ppar-γ*, and negative control were purchase from IGE Biotechnology (Guangzhou, China). pcDNA 3.1, pcDNA 3.1-*Ppar-γ*, pcDNA 3.1-*Slc1a5*, pRL-TK, pGL4.0 and *Slc1a5* luciferase reporters (-1400/+100 bp, -1000/+100 bp and a PPRE-mutated version) were obtained from Kaile Technologies (Guangzhou, China).

### Mice

Male wild-type C57BL/6 mice were obtained from HFK Biotechnology (Beijing, China). Male *leptin*-deficient (*ob/ob*) mice (on a C57BL/6 background) were purchased from GemPharmatech (Nanjing, China). Mice were housed in a specific pathogen-free facility under a 12 h light/12 h dark cycle with free access to water and food. Diets were normal chow (D12450J, Research Diets, New Brunswick, NJ) and HFD (60% calories from fat, D12492, Research Diets, New Brunswick, NJ). The procedures for animal experiments were approved by the Institutional Animal Care and Use Committee of Guangzhou University of Chinese Medicine.

### Obese models

Eight-week-old *ob/ob* mice (maintained on a chow diet) were used as one of two obese models. The *ob/ob* and control wild-type mice were kept under constant darkness one day prior to sample collection. Mice (*n* = 5 per time point) were sacrificed at 6 circadian times (CT2, CT6, CT10, CT14, CT18 and CT22), and the SCN, serum, liver and perigonadal WAT samples were collected for gene, protein and/or metabolite analyses. For the second model, 4-week-old wild-type mice were fed HFD for 10 weeks to induce obesity, and switched to chow diet feeding for another 1 week. Control group of mice were kept on the chow diet. The two groups of mice were kept under constant darkness one day prior to sample collection. Samples were collected at a 4-h interval around the clock as described above for *ob/ob* mice. To evaluate the effect of adipocyte *Bmal1* on obesity, wild-type mice were fed HFD for 10 weeks. After 8 weeks, mice were treated with AAV8.*aP2.Bmal1* (2.5*10^11^ virus particles per mouse, Viraltherapy Technologies, Wuhan, China) or control vector via intraperitoneal injection at CT6. 4 weeks later, mice were sacrificed, and serum and WAT were collected for biochemical and expression analyses.

### Human specimens

Omental fat was obtained from control subjects with a BMI of 18.5-23.9 kg/m^2^ and from obese subjects with a BMI of 32-60 kg/m^2^ through abdominal surgery (information provided in Table S1). Individuals with a history of malignancy, chronic inflammatory diseases such as ulcerative colitis and Crohn's disease, active infectious diseases, and drug or alcohol abuse were excluded. Fresh tissue samples were snap-frozen in liquid nitrogen and stored at -80℃ until RNA, protein and metabolite analyses. The individual samples for each group were pooled for Western blotting as the sample amount was limited. Research protocol was approved by the Medical Ethical Committee of the First Affiliated Hospital of Jinan University. An informed consent was obtained from every participant prior to the study.

### H&E staining

Tissues were formalin-fixed and embedded in paraffin. 5 μm paraffin-embedded sections were stained with haematoxylin & eosin (H&E). Images were captured using a AXIO Imager M1 microscope (Carl Zeiss, Oberkochen, Germany). Adipocyte size was quantified using the ImageJ software based on six sections per mouse and five mice per group.

### Biochemical analyses

Triglycerides and cholesterol in serum samples were measured using the biochemical kits (Jiancheng Bioengineering Institute, Nanjing, China). SAM and acetyl-CoA were measured by using ELISA kits (Meimian Biotechnology, Jiangsu, China).

### Quantitative polymerase chain reaction (qPCR)

RNA extraction, cDNA synthesis, and qPCR reactions were performed as described previously 80. Data were normalized to the housekeeping gene (mouse* Gapdh* or human *GAPDH*). Primers are listed in Table S2.

### Western blotting

Protein samples were separated by sodium dodecyl sulfate-polyacrylamide gel electrophoresis (10% or 12% gels) and transferred to a polyvinylidene difluoride membrane. The membrane was sequentially incubated with primary antibodies and a horseradish peroxidase-conjugated secondary antibody. Protein bands were visualized by using enhanced chemiluminescence and Omega Lum G imaging system (Aplegen, Pleasanton, CA). Protein levels were quantified with Fluorchem 5500 software (Fisher Scientific, Fair Lawn, NJ) and normalized to GAPDH or Histone H3 (Figure S14).

### Immunofluorescence staining

Adipose tissues were fixed in 4% paraformaldehyde, and then transferred to a grade series of sucrose solution (10%, 20% and 30%). The sections (6 μm thickness) were blocked with 5% BSA and 0.5% Triton X-100 in phosphate-buffered saline (PBS), and then incubated with anti-BMAL1 antibody. After washing with PBS, sections were incubated with Alexa Fluor 488-conjugated anti-rabbit secondary antibody. Sections were washed, mounted and imaged using a laser scanning microscope (Carl Zeiss, Oberkochen, Germany).

### Locomotor activity analysis

Mice were individually housed in running wheel cages (Lafayette Instrument, Lafayette, IN) that were placed in light-tight cabinets under a 12 h light/dark cycle. After acclimation to the system, mice were subjected to 3 weeks of continuous recording (1 week under a 12 h light/dark cycle and 2 weeks under constant darkness). Locomotor activity was analyzed using the ClockLab software (Actimetrics, Wilmette, IL). The data were separated, pooled and averaged on the basis of light/dark cycle.

### Metabolic cages

Mice were individually housed in Promethion Metabolic Cages (Sable Systems, Las Vegas, NV) for one week before data collection. Indirect calorimetric and energy balance parameters including VO_2_ (oxygen consumption, in l/min), VCO_2_ (carbon dioxide expiration, in l/min) and food intake were assessed for four days. Energy expenditure (EE, in kJ/min) was calculated by using the Weir's equation (16.3 ×VO_2_ + 4.6 ×VCO_2_). Values of EE were normalized to the body weight raised to the power 0.75.

### Metabolomics

Metabolites in obese and control WAT were extracted with 50% methanol. After centrifugation at 4000 g for 20 min, the supernatant was subjected to analysis by a UHPLC‐QTOF/MS system consisting of an ExionLC UHPLC and a TripleTOF 5600^+^ mass spectrometer (AB SCIEX, Warrington, UK). Chromatographic separation was performed with an ACQUITY UPLC T3 column (100*2.1 mm, 1.8 μm, Waters, Milford, MA). The mobile phases consisted of 0.1% formic acid (phase A) and acetonitrile in 0.1% formic acid (phase B). The gradient elution program was 5% phase B for 0-0.5 min, 5-100% phase B for 0.5-7 min, 100% phase B for 7-8 min, 100-5% phase B for 8-8.1 min, and 5% phase B for 8.1-10 min. The flow rate was 0.4 ml/min. The capillary voltage was set at 5 kV in positive mode and -4.5 kV in negative mode. LC-MS raw data file were converted into a common format (.mzdata), followed by data processing (including peak picking, peak grouping and retention time correction) with the XCMS software. The HMDB and KEGG databases were used to annotate the metabolites. Metabolite quantitation and differential metabolite identification were performed using metaX software. Differential metabolites between obese and normal WAT were defined as such when Benjamini-Hochberg-adjusted p-value was < 0.05. KEGG pathways fulfilling the criterion of a hypergeometric p < 0.05 were defined as significantly enriched in differential metabolites.

### ChIP

ChIP assays were performed using a SimpleChIP plus Enzymatic Chromatin IP kit (Cell Signaling Technology, Beverly, MA) as described in our previous publication 79. Briefly, WAT was fixed with formaldehyde, followed by the addition of glycine. Sample was lysed and chromatin was sheared (to a size of 150-900 bp) using the micrococcal nuclease. Sheared chromatin was subjected to immunoprecipitation with anti-H3K4me3, anti-H3K27ac, anti-PPAR-γ or normal IgG, followed by incubation with protein G magnetic beads for 2 h. The immune complex was eluted to collect DNA fragments, and DNA fragments were purified using spin columns. The abundance of precipitated DNA fragments was determined by qPCR using the primers for targeted loci (Table S3).

### Histone acetylation and methylation inhibition assays

3T3-L1 adipocytes (3T3-L1 cells were differentiated into adipocytes by treatment with 3-isobutyl-1-methylxanthine, dexamethasone, and insulin) were cultured in Dulbecco's modified Eagle's medium (DMEM). For histone acetylation inhibition, cells were pretreated with C646 (final concentration: 2, 5 or 10 μM) or vehicle for 12 h, and treated with 10 mM glutamine for another 24 h. For histone methylation inhibition, cells were pretreated with MM-102 (final concentration: 5, 20 or 50 μM) or vehicle for 12 h, and treated with 30 μM methionine for another 24 h. At the end of the experiments, cells were harvested for RNA and protein extraction.

### Cell synchronization

Cell synchronization (serum shock) was performed as previously described 81. Briefly, 3T3-L1 adipocytes were cultured in DMEM containing 10% fetal bovine serum (FBS, Gibco, Waltham, MA). On the next day, the culture medium was replaced with a serum-free medium. 12-h later, 50% FBS was added for 2 h. The medium was then changed back to serum-free medium containing 2 mM glutamine, 30 μM methionine, 1 μM rosiglitazone or vehicle. Cells were harvested for RNA extraction and qPCR at 0, 4, 8, 12, 16, 20 and 24 h after synchronization.

### Real-time luminescence monitoring

U2OS cells stably overexpressed with *BMAL1*-*Luc* (a *BMAL1* promoter-driven luciferase reporter) were used to assess transcription activity of* BMAL1*. U2OS cells were seeded into 35 mm dishes and maintained in DMEM containing 10% FBS. On next day, cells were pre-treated with glutamine- or methionine-free DMEM for 24 h, followed by incubation with a recording medium (10 mM HEPES, 3.5 g/l glucose, 0.35 g/l NaHCO_3_, 1% penicillin-streptomycin, 10% FBS, 0.1 mM luciferin and 100 nM dexamethasone in DMEM) containing 10 mM glutamine or 30 μM methionine. Luminescence data (counts/s) were collected by using a LumiCycle high-throughput luminometer (Actimetrics, Wilmette, IL).

### Glutamine and methionine treatment

Four-week-old wild-type mice were fed on HFD for 6 weeks. The mice continued on HFD and we initiated feeding of glutamine (1%, in drinking water) or methionine (0.3%, in drinking water) or pure drinking water for 4 weeks. Body weight was monitored weekly. Mice were sacrificed at CT6, and serum and WAT samples were collected for further experiments. We also performed short-term treatment experiments. Four-week-old wild-type mice were fed on HFD or chow diet for 8 weeks. The mice continued on HFD or chow and we initiated feeding of glutamine (1%, in drinking water) or methionine (0.3%, in drinking water) or pure drinking water for 2 weeks. Body weight was monitored and samples were collected as described above.

### Luciferase reporter assay

Luciferase reporter assays were performed as previously described 79. Briefly, 3T3-L1 adipocytes were co-transfected with *Slc1a5* luciferase reporter (-1400/+100, -1000/+100 bp or PPRE-mutated version) and pRL-TK vector using a JetPRIME transfection kit. 24 h later, the culture medium was replaced with fresh medium containing 1 μM rosiglitazone (a PPAR-γ agonist) or vehicle. After another 24 h, cells were lysed, and luciferase activities were determined by using Dual-Luciferase Reporter Assay System (Promega, Madison, WI). Luciferase activity was expressed as relative luciferase unit (RLU).

### [^13^C_5_]-glutamine and [^2^H_3_]-methionine uptake assays

Cellular uptake assays were performed with [^13^C_5_]-glutamine or [^2^H_3_]-methionine following a published protocol 82. Briefly, 3T3-L1 adipocytes were seeded into 6-well plates and maintained in DMEM containing 10% FBS. Cells were treated with rosiglitazone or siSlc1a5 or control for 24 h with or without following treatment with *Slc1a5* plasmid for 24 h. Cells were washed with PBS and incubated with 2 ml isotope-containing medium (glutamine-free DMEM supplemented with 60 μM [^13^C_5_]-glutamine or methionine-free DMEM supplemented with 200 μM [^2^H_3_]-methionine) for 1 h. Cells were solubilized in 80% methanol. After centrifugation at 14000 g for 20 min, supernatant was collected and dried under nitrogen gas. Residues were resuspended in 80% methanol and subjected to UPLC-QTOF/MS analyses.

### UPLC-QTOF/MS analysis

Glutamine and methionine (including [^13^C_5_]-glutamine and [^2^H_3_]-methionine) were quantified using a Waters UPLC-QTOF/MS system consisting an ACQUITY UPLC and a Xevo G2 QTOF mass spectrometer. Chromatographic separation was performed using a BEH Amide column (1.7 μm, 2.1 x 100 mm) (Waters, Milford, MA). The mobile phase consisted of 20 mM ammonium acetate in water (phase A) and acetonitrile in 0.1% formic acid (phase B). The flow rate was 0.2 ml/min. The gradient elution program was 5% phase B for 0-1 min, 5-10% phase B for 1-2 min, 10-50% phase B for 2-3.5 min, 50-95% phase B for 3.5-4 min, 95% phase B for 4-4.5 min, and 95-5% phase B for 4.5-5 min. The mass spectrometer was operated at the positive ion mode.

### Statistical analyses

Data are recorded as mean ± standard deviation (SD). Means of two groups were compared using Student's t-test. Analysis of variance (ANOVA, one-way or two-way) was performed to compare means of more than two groups. In most cases, we performed two-way ANOVA to analyze whether the effect of obesity was significant or not. If so, post-hoc Bonferroni test was used for further group comparisons. The level of significance was set at p < 0.05 (*).

## Supplementary Material

Supplementary figures and tables.

## Abbreviations

AAV8: adeno-associated virus serotype 8
Ac-CoA: acetyl coenzyme A
BHMT: betaine homocysteine methyltransferase
BMAL1: brain and muscle ARNT-like protein-1
CLOCK: circadian locomotor output cycles kaput
ChIP: chromatin immunoprecipitation
CRY: cryptochrome
CT: circadian time
DBP: albumin D-site-binding protein
DMEM: Dulbecco's Modified Eagle's Medium
E4BP4: E4 promoter-binding protein 4
EE: energy expenditure
FBS: fetal bovine serum
Gln: glutamine
GLUL: glutamine synthetase
GLS: glutaminase
H3K4: histone H3 lysine 4
H3K27: histone H3 lysine 27
HFD: high fat diet
Met: methionine
NPAS2: neuronal PAS domain protein 2
MAT2A: methionine adenosyltransferase II, alpha
MTR: 5-methyltetrahydrofolate-homocysteine methyltransferase
PER: period
qPCR: quantitative polymerase chain reaction
PPAR: peroxisome proliferator-activated receptor
PPRE: PPAR response element
ROR: RAR-related orphan receptor
SAM: S-adenosylmethionine
SLC: solute carrier
WAT: white adipose tissue
