# Supplementary material for: PPAR-γ integrates obesity and adipocyte clock through epigenetic regulation of Bmal1
Source: Theranostics. 2022 Jan 16;12(4):1589–606. doi: 10.7150/thno.69054 (PMC8825584; doi:10.7150/thno.69054)
Supplement: Supplementary file 1 — Supplementary figures and tables. [file thnov12p1589s1.pdf]

Supplementary Materials for

PPAR-γ integrates obesity and adipocyte clock through epigenetic regulation of *Bmal1*

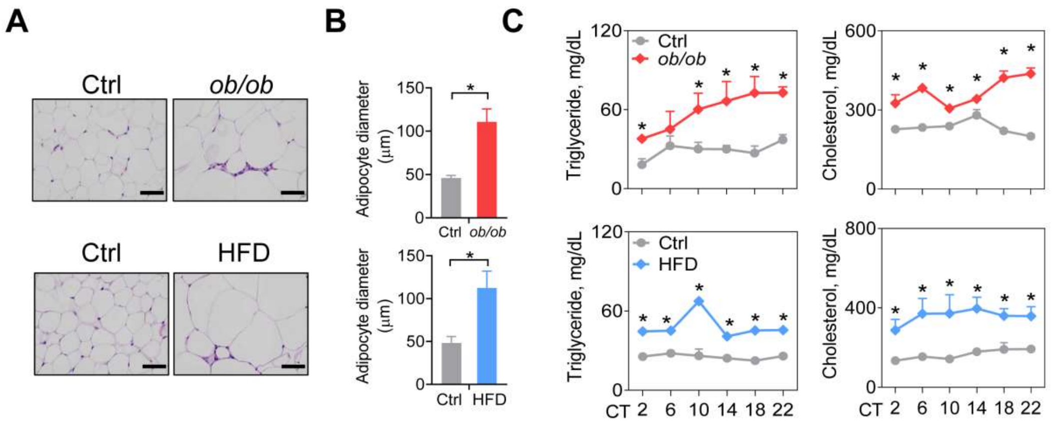

Fig S1. Validation of two obese models. (A) H&E staining of WAT derived from *ob/ob*, HFD and control mice. Scale bar: 50 μm. **(B)** The diameter of adipocytes in WAT from *ob/ob*, HFD and control mice. Data are mean ± SD (n = 5). *p < 0.05 (t test). **(C)** Serum triglyceride and cholesterol levels in *ob/ob*, HFD and control mice at 6 circadian time points. Data are mean ± SD (n = 5). *p < 0.05 at individual time points as determined by two-way ANOVA and Bonferroni post hoc test.

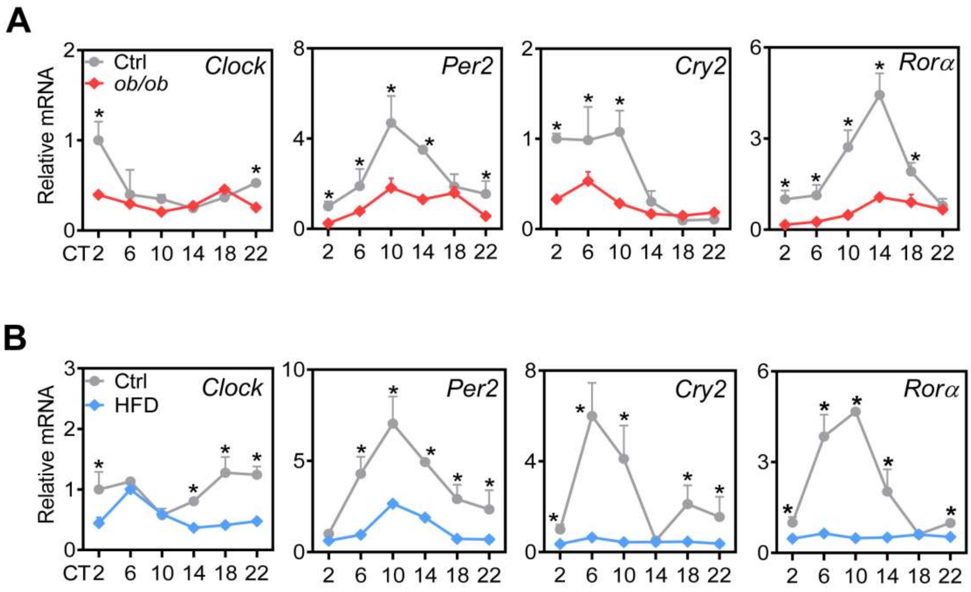

Fig S2. Disruption of adipocyte clock in obese mice. (A and B) mRNA expression of clock genes (*Clock*, *Per2*, *Cry2* and *Ror*α) in WAT derived from *ob/ob* **(A)**, HFD **(B)** and control mice at 6 circadian times. Data are mean ± SD (n = 5). *$p < 0.05$ at individual time points as determined by two-way ANOVA and Bonferroni post hoc test.

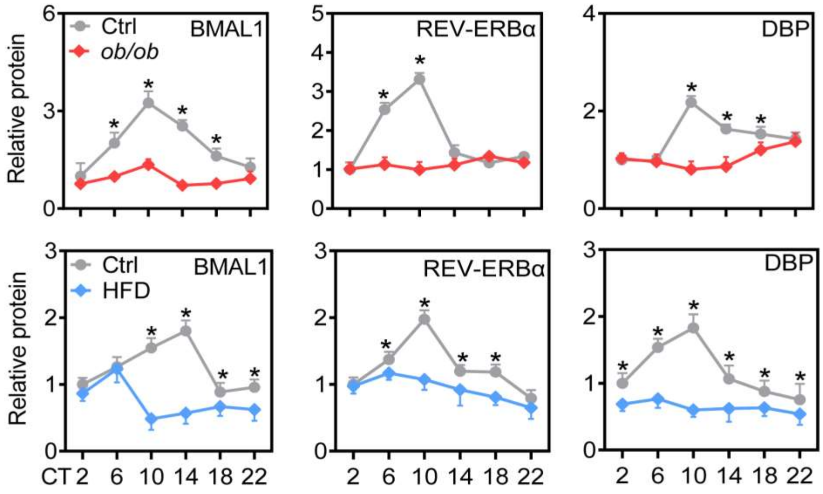

Fig S3. Quantification data on the protein bands in Fig 1D. Data are mean ± SD (*n* = 5). *p < 0.05 as determined by two-way ANOVA followed by Bonferroni post hoc test.

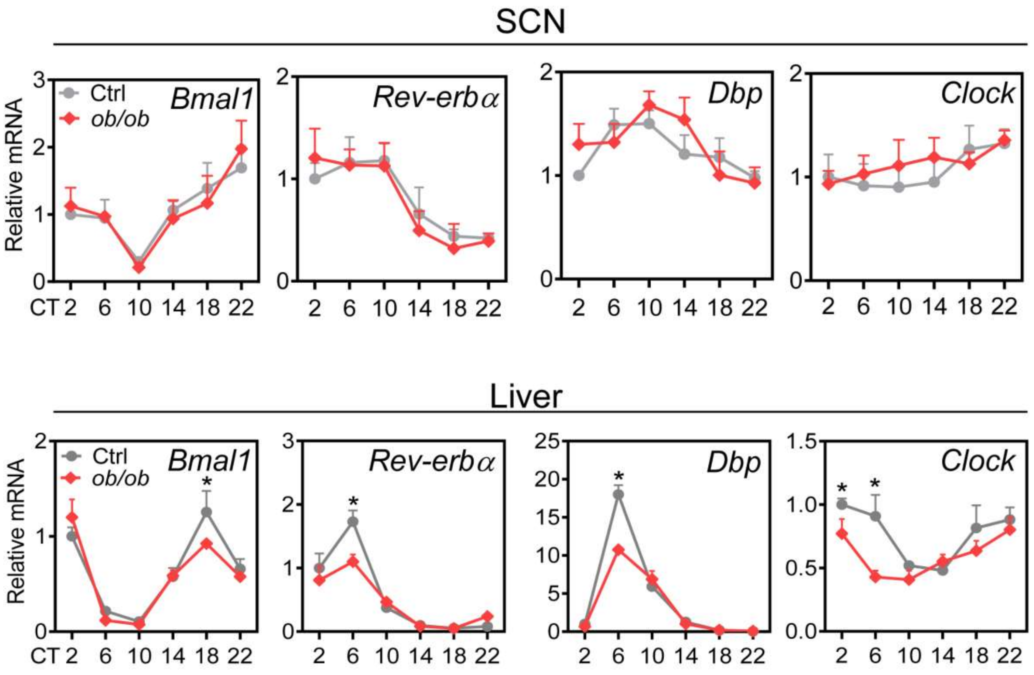

Fig S4. mRNA expression of clock genes in SCN and liver of obese mice. mRNA expression of clock genes (*Bmal1*, *Rev-erbα*, *Dbp* and *Clock*) in SCN (upper panel) and liver (lower panel) derived from *ob/ob* and control mice at 6 circadian times. Data are mean ± SD ($n = 5$). *$p < 0.05$ at individual time points as determined by two-way ANOVA and Bonferroni post hoc test.

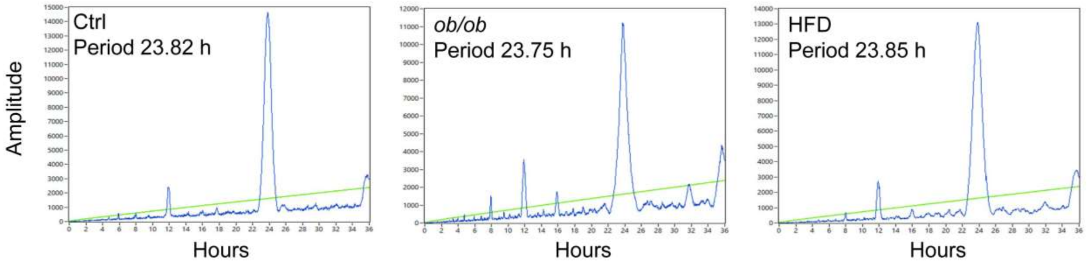

Fig S5. Free-running periods of *ob/ob*, HFD and control mice.

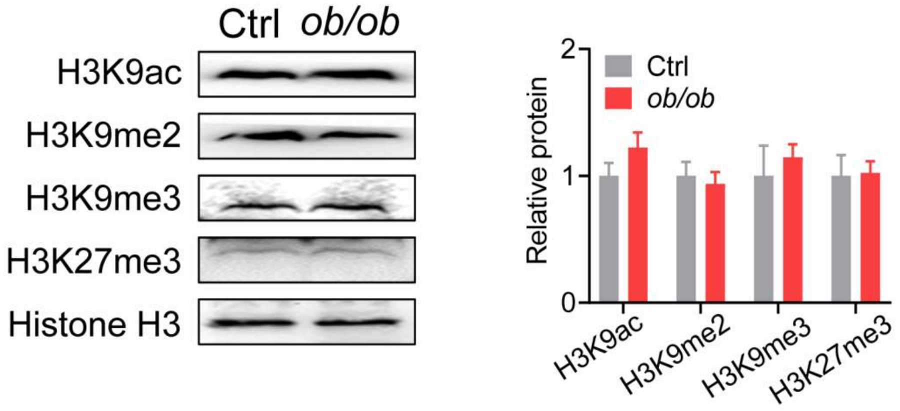

Fig S6. Protein expression of H3K9ac, H3K9me2, H3K9me3 and H3K27me3 in WAT tissues of *ob/ob* and control mice at CT6. Data are mean ± SD (*n* = 5).

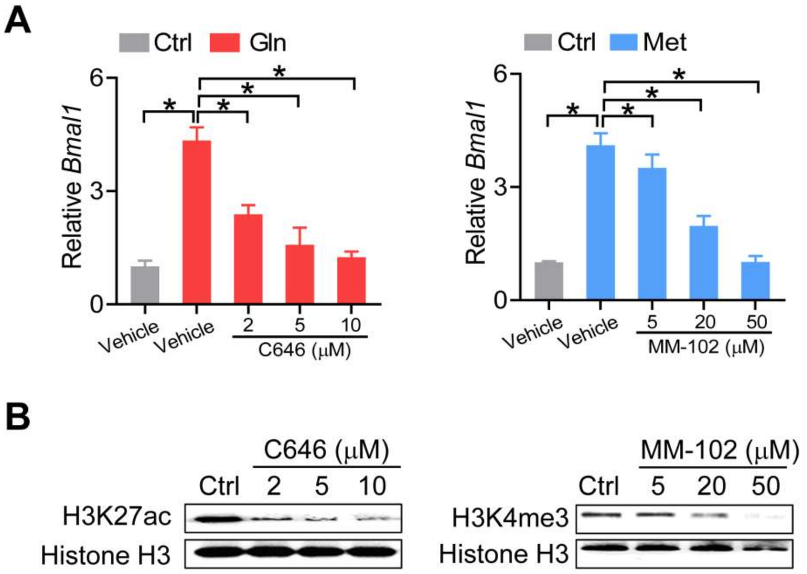

Fig S7. Effects of C646 and MM-102 on the expression of *Bmal1* (A), H3K27ac and H3K4me3 (B) in 3T3-L1 adipocytes. Data are mean ± SD (n = 3). *$p < 0.05$ (one-way ANOVA and Bonferroni post hoc test).

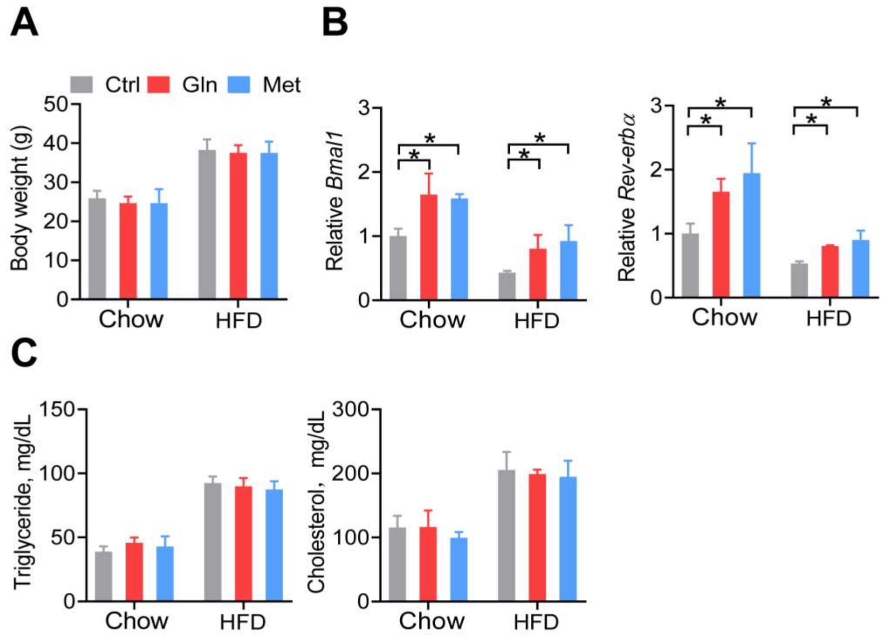

Fig S8. Short-term (two weeks) treatment of glutamine or methionine enhances *Bmal1* expression independent of body weight. (A) Body weight of HFD- and chow-fed mice treated with glutamine or methionine. **(B)** mRNA expression of *Bmal1* and *Rev-erbα* in WAT in HFD- and chow-fed mice treated with glutamine or methionine. **(C)** Serum triglycerides and cholesterol in HFD- and chow-fed mice treated with glutamine or methionine. All data are mean ± SD (n = 5). *$p < 0.05$ (one-way ANOVA and Bonferroni post hoc test).

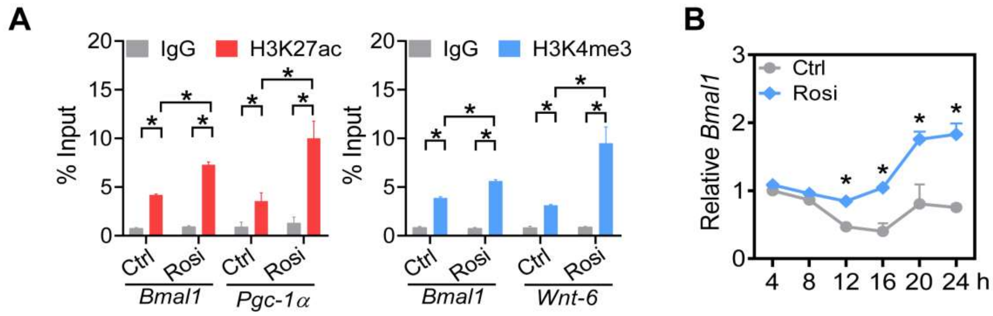

Fig S9. Effects of rosiglitazone (Rosi) on enrichments of H3K27ac and H3K4me3 at the *Bmal1* promoter (A) and on *Bmal1* expression (B) in 3T3-L1 adipocytes. Data are mean ± SD (n = 3). *$p < 0.05$ (two-way ANOVA and Bonferroni post hoc test).

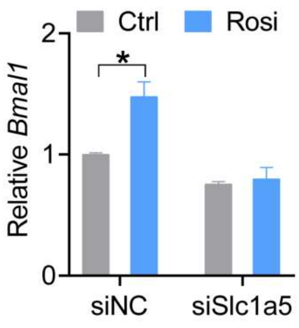

Fig S10. Effects of rosiglitazone (Rosi) on mRNA expression of *Bmal1* in 3T3-L1 adipocytes transfected with siSlc1a5 or control (siNC). Data are mean ± SD (n = 3). *$p < 0.05$ (t-test).

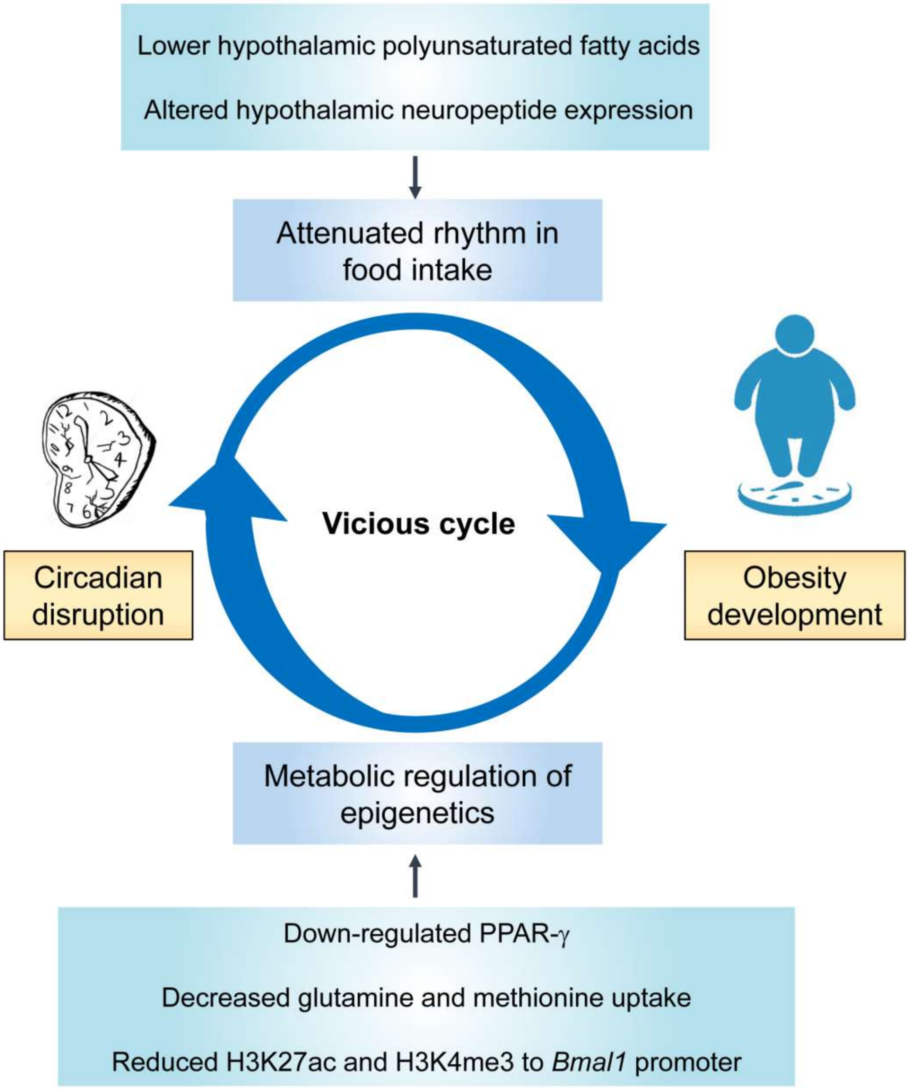

Fig S11. Schematic diagram showing a vicious circle between circadian disruption and obesity development.

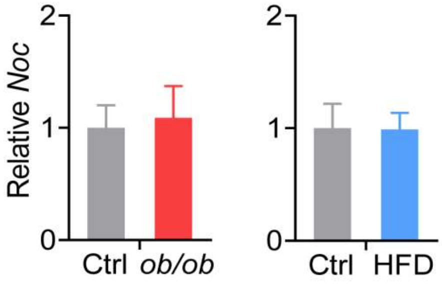

Fig S12. mRNA expression of *nocturnin* (Noc) in WAT tissues in HFD-induced obese and control mice. Data are mean ± SD (*n* = 5).

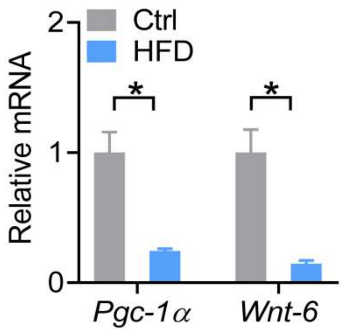

Fig S13. mRNA expression of non-clock genes (*Pgc-1α* and *Wnt-6*) in WAT tissues in HFD-induced obese and control mice. Data are mean ± SD (n = 5). *$p < 0.05$ (t test).

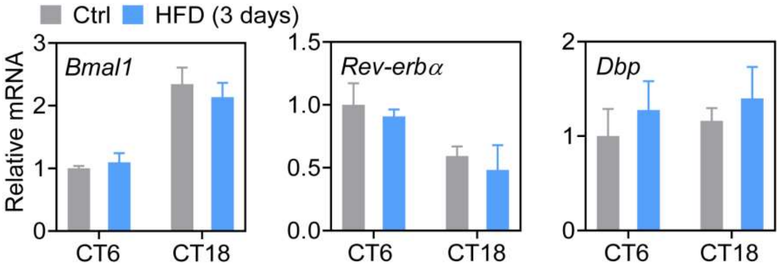

Fig S14. Effects of HFD feeding for 3 days on expression of clock genes (*Bmal1*, *Rev-erb*α and *Dbp*) in WAT. Data are mean ± SD (*n* = 5).

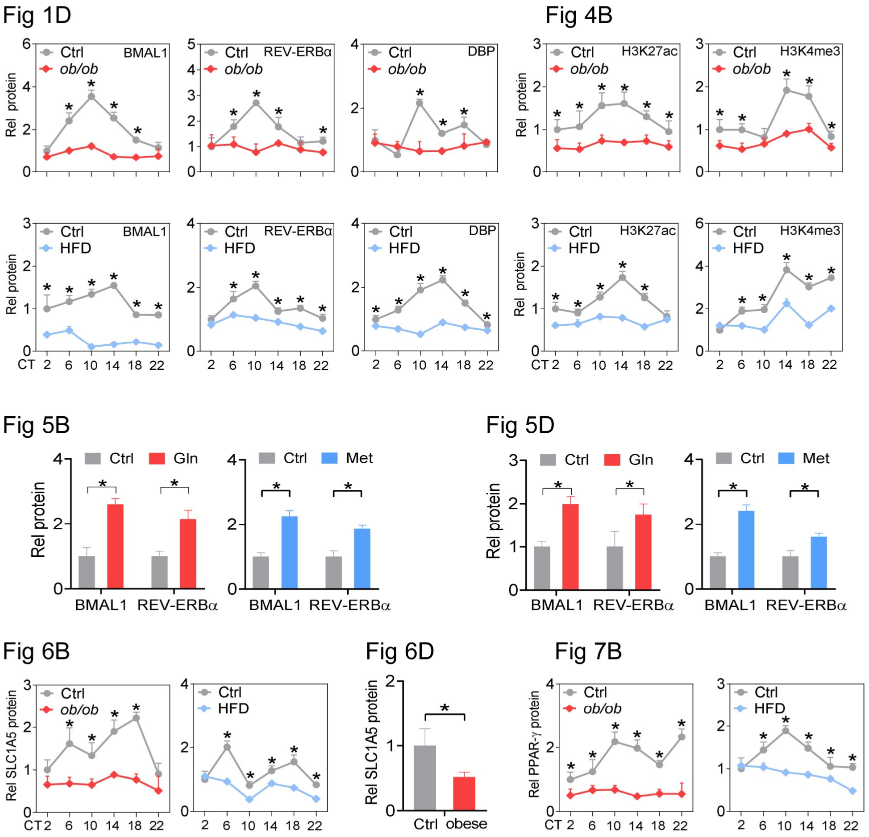

Figure S15. Quantification data generated from Western blots in this study. Data are mean ± SD. *$p < 0.05$.

Table S1. Characteristics of obese and lean subjects.

**Subjects**	**Obese**	**Lean**
Number	13	10
Age	28.5 ± 5.1	35.4 ± 14.6
Gender (F/M)	10/3	6/4
BMI	40.2 ± 7.1	20.8 ± 1.8

BMI, body mass index; F, female; M, male.

Table S2. Sequences of primers for qPCR analysis

Name	Forward (5'-3' sequence)	Reverse (5'-3' sequence)
mBmal1	CTCCAGGAGGCAAGAAGATTC	ATAGTCCAGTGGAAGGAATG
mClock	CCAGAGGGAGAACATTCA	TGGCTCCTTTGGGTCTAT
mRev-erbα	TTTTTCGCCGGAGCATCCAA	ATCTCGGCAAGCATCCGTTG
mRev-erbβ	GGAGTTCATGCTTGTGAAGGCT	CAGACACTTCTTAAAGCGGCAC
mPer1	GAAAGAAACCTCTGGCTGTTCC	GCTGACGACGGATCTTTCTTG
mPer2	CCACACTTGCCTCCGAAATA	ACTGCCTCTGGACTGGAAGA
mCry1	CCCAGGCTTTTCAAGGAATGGA	GCAGGGAGTTTGCATTCATTCG
mCry2	GATGCCGATTTCAGTGTGAATG	GGCAGTAGCAGTGGAAGAAT
mDbp	ACATCTAGGGACACACCCAGTC	AAGTCTCATGGCCTGGAATG
mRorα	GAGACCCCGCTGACCCA	TGACTGAGATACCTCGGCTG
mSlc1a5	CATGTAAAATACCGCAATCCTGT	GACGATAGCGAAGACCACCA
mSlc38a1	ATCTTCGGAGCCACCTCTCT	TGCATCCTCCTCTCCCATGA
mSlc38a2	CATCCCGCTGTTCTTCCCAT	AAAGAGAGCAGCGAGCAAGT
mBhmt	TTAGAACGCTTAAATGCCGGAG	GATGAAGCTGACGAACTGCCT
mMtr	TCCTCCTCGGCCTATCTTTATTT	GGTCCGAATGAGACACGCT
mMat2a	GGTCATTGTCAGGGATCTGGA	ACCAAAGTGGCCATAGGCTG
mGlul	AGGCACCAGTACCACATTCG	CACCGGCAGAAAAGTCGTTG
mGls	GCAGCGGGATTATGACTCCA	ATTCCACCTGTCCTTGGGGA
mGls2	AGTTCACCACGGCTCTGAAG	TGCTGCTCACACACTTTTGG
mPpar-γ	AACCCACAACCAAATCCACAC	ATCACGGAGAGGTCCACAGA
mPgc-1α	GCACGCAGCCCTATTCATTG	TGAGTCTCGACACGGAGAGT
mWnt-6	GCAGGACATCCGAGAGACAG	TGGAACAGGCTTGAGTGACC
mGapdh	CAAGGAGTAAGAAACCCTGGA	CGAGTTGGGATAGGGCCTCT
mNoc	CGCGTCCTGGTGCCTAATCT	GAGCGCGTGTCTGGGAGAA
hBMAL1	ACTTCCCCTCTACCTGCTCA	ATCCAGCCCCATCTTTGTGG
hREV-ERB	GACATGACGACCCTGGACTC	GCTGCCATTGGAGTTGTCAC
hDBP	CCGCTATCTTTCTATTAACTGAC	TCAACCAGCTACAAAAAGCATG
hSLC1A5	AGGCTTTCTCTGGCTGGTAA	ACCCAGGCTCTTAGGTCCG
hGAPDH	CATGAGAAGTATGACAACAGCC	AGTCCTTCCACGATACCAAAGT

m: mouse h: human

Table S3. Sequences of primers for ChIP assays

Name	Forward (5'-3' sequence)	Reverse (5'-3' sequence)
*Bmal1*_H3K27ac	GTAGGTCAGGGACGGAGGT	GCAGCCATGCCGACACTCA
*Pgc-1α*_H3K27ac	CAAAGCTGGCTTCAGTCAC	AAAAGTAGGCTGGGCTGTCA
*Bmal1*_H3K4me3	AGAGATGCGGCGTTTTCTC	GTGACTGCCTCTCAGCTCTC
*Wnt-6*_ H3K4me3	CTTCCTTCCCCCAAAGAAAT	GTCCAACAGCTCTTCCCTACCTATC
*Slc1a5*_PPRE	TTCCTTCTCCAACAAGCCCT	GTCTGTTCTACCAGGCGCTC
